# Biopolymer-Based Nanocomposite Edible Coatings for Postharvest Preservation of Horticultural Crops: Mechanisms, Functional Additives, and Safety Perspectives

**DOI:** 10.3390/polym18212680

**Published:** 2026-10-28

**Authors:** Mukhtar Iderawumi Abdulraheem, Yusuf Ola Mukaila, Marissa Pérez-Alvarez, Gregorio Cadenas-Pliego, Olaniyi Amos Fawole

**Affiliations:** 1 Postharvest and Agroprocessing Research Centre, Department of Botany and Plant Biotechnology, University of Johannesburg, Auckland Park, Johannesburg 2006, South Africa; abiduct2020@gmail.com; 2 South African Research Chairs Initiative in Sustainable Preservation and Agroprocessing Research, Faculty of Science, University of Johannesburg, Auckland Park, Johannesburg 2006, South Africa; 3 Centro de Investigación en Química Aplicada, Blvd. Enrique Reyna 140, Saltillo 25294, Coahuila, Mexico; marissa.perez@ciqa.edu.mx

**Keywords:** edible coatings, biopolymers, nanocomposites, postharvest preservation, horticultural crops, food safety, functional additives

## Abstract

Postharvest losses of horticultural crops represent a significant global challenge, impacting food security, economic stability, and environmental sustainability. Traditional preservation methods cannot maintain the quality and extend the shelf life of these highly perishable commodities. In response, biopolymer-based nanocomposite edible coatings have emerged as a promising, eco-friendly alternative. Existing reviews typically address barrier mechanisms, functional additives, or safety in isolation and rarely integrate nanomaterial migration and regulatory considerations with coating design and application. This gap limits the translation of laboratory findings into safe, commercially viable coatings. This review therefore provides an integrated, mechanism-to-safety synthesis of biopolymer nanocomposite edible coating. This review examines the physiological drivers of postharvest deterioration, and the barrier functions through which coating counteract them. The review further evaluates polysaccharide, protein and lipid matrices, the nanomaterials used as fillers and active agents, and the functional additives that confer antimicrobial and antioxidant activity. Conventional and emerging application methods are compared, and their effects on physicochemical and sensory quality are assessed. The novelty of this work lies in three contributions: (i) an integrated framework linking coating mechanisms, formulation and application to safety outcomes; (ii) a dedicated treatment of nanoparticle migration, FDA/EFSA regulatory status and consumer perception, areas that are only briefly mentioned in comparable reviews; and (iii) a systematic comparative analysis against recent reviews, which clarifies where this synthesis advances the field. Remaining challenges (industrial scalability, cost-effectiveness and integration with smart packaging) are identified, and research priorities are prosed to support the development of safe, effective and sustainable coatings that reduce food waste and improve food quality. The practical impact of this synthesis is that it provides formulators with a trade-off framework that identifies which coating properties can be optimized simultaneously, and it gives regulators and industry a consolidated account of the migration, toxicity and regulatory evidence needed before commercial adoption.

## 1. Introduction

Horticultural crops, encompassing fruits, vegetables, and ornamental plants, are indispensable components of the global food system and human diet, providing essential vitamins, minerals, dietary fiber, and antioxidants. Their cultivation and trade contribute significantly to agricultural economies worldwide. However, these commodities are inherently perishable, characterized by high metabolic activity, rapid senescence, and susceptibility to microbial spoilage and physical damage. Consequently, substantial postharvest losses occur along the entire supply chain, from harvesting and handling to storage, transportation, and retail. Global estimates suggest that a considerable percentage, often ranging from 30% to 50%, of horticultural produce is lost before reaching consumers, leading to immense economic losses, food insecurity, and environmental burdens due to wasted resources and greenhouse gas emissions [1,2].

The challenges in postharvest preservation are multifaceted, primarily stemming from the physiological processes inherent to living plant tissues. Respiration, transpiration, and ethylene production continue after harvest, leading to moisture loss, weight reduction, softening, color changes, and nutrient degradation [3]. Moreover, horticultural crops are highly vulnerable to attack by various pathogenic microorganisms, including bacteria and fungi, which cause rot and decay, further accelerating spoilage [4,5]. Traditional preservation strategies, such as refrigeration, controlled atmosphere storage, and chemical treatments, have been widely employed to mitigate these losses. While effective to some extent, these methods often present limitations, including high energy consumption, environmental concerns associated with synthetic chemicals, and potential health risks from residues [6]. Furthermore, the increasing consumer demand for fresh, minimally processed, and chemical-free produce necessitates the development of innovative, safe, and sustainable preservation technologies.

In recent decades, edible coatings have emerged as a promising, eco-friendly alternative to conventional packaging and chemical preservatives. Edible coatings are thin layers of food-grade materials applied directly to the surface of horticultural products, forming a protective barrier that can regulate gas exchange, reduce moisture migration, and carry active compounds [7]. These coatings are typically composed of natural biopolymers, which are biodegradable, biocompatible, and often derived from renewable resources, aligning with the principles of a circular bioeconomy [8]. The concept of edible coating is not new, with historical applications dating back centuries. However, significant advancements in materials science and nanotechnology have revolutionized their design and functionality, leading to the development of biopolymer-based edible nanocomposite coatings.

The integration of nanomaterials into biopolymer matrices has enhanced the performance of edible coatings. The three nanomaterial classes used in coatings behave differently in ways that matter for both function and safety, so claims of functional enhancement are specified by class throughout this review. Inorganic nanoparticles (AgNPs, ZnONPs, TiO_2_NPs, CuONPs) provide intrinsic antimicrobial activity through ion release and reactive oxygen species generation, but are also the class with demonstrated migration and toxicity concerns. Organic nanofillers (cellulose nanocrystals, chitin whiskers) improve mechanical and barrier performance without intrinsic antimicrobial activity and are generally regarded as lower-hazard. Nanoemulsions and nanocapsules are delivery systems whose behaviour depends on the encapsulated payload and encapsulation efficiency rather than on the carrier itself [9,10]. These nanocomposites create a more effective protective layer, slowing down ripening, reducing microbial growth, and preserving the sensory and nutritional quality of horticultural crops for extended periods [11]. The ability to tailor the properties of these coatings by selecting specific biopolymers, nanomaterials, and functional additives offers a versatile platform for addressing the diverse preservation needs of various fruits and vegetables.

This review aims to provide a detailed and comprehensive overview of the current state-of-the-art in biopolymer-based nanocomposite edible coatings for postharvest preservation of horticultural crops. We explored the fundamental mechanisms by which these coatings exert their protective effects, delving into gas barrier properties, moisture regulation, and antimicrobial and antioxidant activities. A dedicated section discussed the various functional additives that can be incorporated to enhance coating performance, along with their synergistic effects. Furthermore, the manuscript addressed the critical aspects of safety, regulatory frameworks, and toxicological considerations associated with the use of nanomaterials in food applications. We also examined the different application methods and their impact on the physicochemical and sensory attributes of coated produce. To provide a holistic perspective, this review included a comparative analysis of recent review papers in this field, highlighting the novelty and unique contributions of the current work. By synthesizing the latest scientific advancements and identifying future research directions, this paper serves as a valuable resource for researchers, industry professionals, and policymakers working towards sustainable postharvest solutions and reduced food waste.

## 2. Literature Search and Selection

The review was conducted as a narrative review with a structured and reproducible search strategy. The methodology was designed to capture recent advances in biopolymer-based nanocomposite edible coatings while ensuring that foundational studies were not excluded.

### 2.1. Databases and Search Strings

Literature was identified through systematic searches of Scopus, Web of Science Core Collection, PubMed, and ScienceDirect. Google Scholar was used for forward citation tracking of key reviews and to identify grey literature, including preprint versions of relevant articles. The search was conducted in March 2026 and covered the period from January 2015 to March 2026, with no language restriction beyond English-language publications. Foundational studies published before 2015 were included only where they established a mechanism or material property not superseded by more recent work.

*The search strings were constructed around three concept blocks combined with the AND operator, with synonyms within each block combined using OR. Block 1 (coating systems) comprised: “edible coating” OR “edible film” OR “biopolymer coating” OR “nanocomposite coating” OR “bio-nanocomposite”. Block 2 (nanomaterials and additives) comprised: nanomaterial OR nanoparticle* OR nanoemulsion* OR “cellulose nanocrystal” *OR “zinc oxide” OR “silver nanoparticle”* OR “titanium dioxide” OR “essential oil” *OR* “*plant extract*”. Block 3 (application context) comprised: postharvest OR “shelf life” OR “fruit preservation” OR “vegetable preservation” OR horticulture OR “fresh produce”. Search terms were adapted to the syntax and controlled vocabulary of each database (e.g., MeSH terms in PubMed).

### 2.2. Inclusion and Exclusion Criteria

Studies were included if they met all of the following criteria: (i) the study addressed edible coatings or films applied to horticultural crops (fruits, vegetables, or ornamental plants) for postharvest preservation; (ii) the coating system incorporated a nanomaterial, nanoemulsion, or nanoencapsulated active agent, or addressed a biopolymer matrix subsequently used in nanocomposite formulations; (iii) the study reported at least one quantitative outcome related to postharvest quality, shelf life, barrier properties, antimicrobial activity, or safety; and (iv) the article was a peer-reviewed original research paper, review, or book chapter published in English. Studies were excluded if they: (i) addressed food packaging applications in which the material was not intended to be consumed (e.g., non-edible films, conventional plastic packaging); (ii) focused on non-horticultural commodities (e.g., cereals, meat, dairy) without relevance to produce preservation; (iii) were conference abstracts, editorials, or opinion pieces without primary data or systematic synthesis; (iv) did not report nanomaterial or nanotechnology components, unless the study provided essential background on biopolymer matrix properties; or (v) were duplicates of records already identified.

### 2.3. Screening Process and Record Numbers

**Figure 1:**
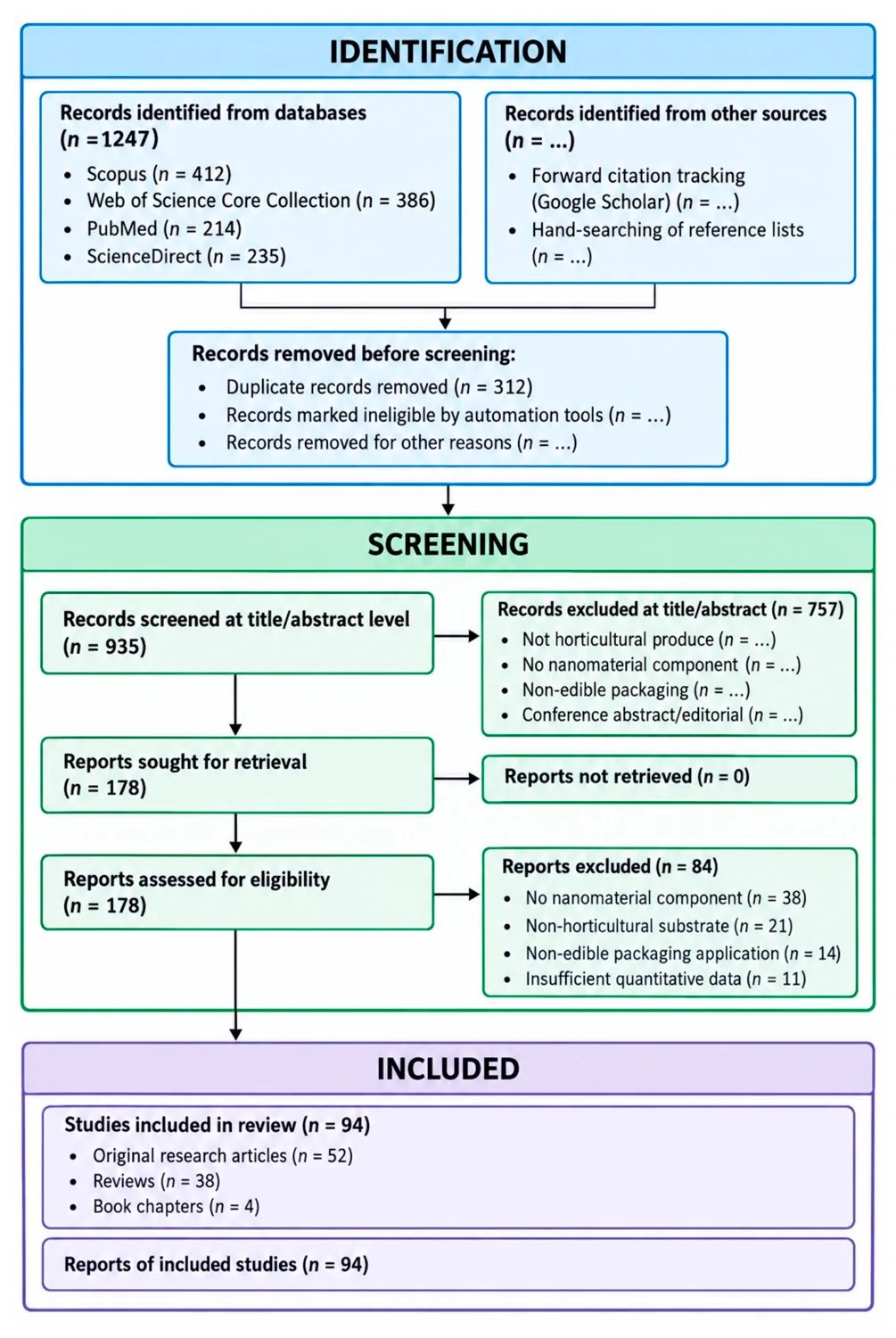
PRISMA 2020 flow diagram of the literature identification, screening, and inclusion process.

Records were exported to Rayyan systematic review software for deduplication and screening. After removal of duplicates, titles and abstracts were screened independently against the inclusion criteria. Disagreements were resolved by discussion, with the supervisor adjudicating where consensus could not be reached. Records retained after title and abstract screening were assessed in full text by the same two reviewers. Reference lists of included reviews were hand-searched for additional eligible studies. The search returned 1247 records across the four databases. After removal of 312 duplicates, 935 records were screened at title and abstract level. Of these, 178 were retained for full-text assessment. Following full-text screening, 94 studies met the inclusion criteria and were included in the review. The principal reasons for exclusion at the full-text stage were: absence of nanomaterial components (38 records), non-horticultural substrate (21 records), non-edible packaging application (14 records), and insufficient quantitative outcome data (11 records). The included studies comprised 52 original research articles, 38 reviews, and 4 book chapters. A PRISMA 2020 flow diagram summarising the identification, screening, and inclusion process is provided as Figure 1.

## 3. Postharvest Physiological Challenges and the Protective Role of Edible Coatings

The postharvest life of horticultural crops is a race against time, dictated by the inherent physiological and biochemical processes that continue after the product is detached from the parent plant. Unlike staple grains, fruits and vegetables are living tissues with high water content and active metabolisms. The primary physiological challenges include respiration, transpiration, and ethylene-mediated ripening, all of which contribute to the eventual senescence and spoilage of the produce [12]. Respiration is the oxidative breakdown of complex organic molecules (such as sugars and organic acids) into simpler molecules (CO_2_ and H_2_O), accompanied by the release of energy and heat. High respiration rates lead to the rapid depletion of energy reserves, accelerated ripening, and increased metabolic heat, which can further stimulate spoilage. Transpiration, the loss of water vapor from the produce surface, is the main cause of weight loss, shriveling, and loss of textural crispness. Ethylene, a gaseous plant hormone, acts as a signaling molecule that triggers and coordinates the ripening process in climacteric fruits, leading to changes in color, flavor, and texture [13].

Edible coatings serve as a multifunctional protective layer that addresses these physiological challenges by creating a modified internal atmosphere and a physical barrier to moisture loss. By acting as a semi-permeable membrane, these coatings can significantly reduce the oxygen (O_2_) concentration and increase the carbon dioxide (CO_2_) concentration within the produce [4]. This modified atmosphere effectively slows down the respiration rate, thereby preserving the energy reserves and delaying the onset of senescence. Furthermore, the barrier properties of the coating against ethylene diffusion can delay the ripening process, providing more time for transport and marketing [14]. The hydrophobic nature of certain coating components, particularly lipids and waxes, creates a barrier that retards transpiration, helping to maintain the turgidity and freshness of the fruits and vegetables [15].

**Figure 2:**
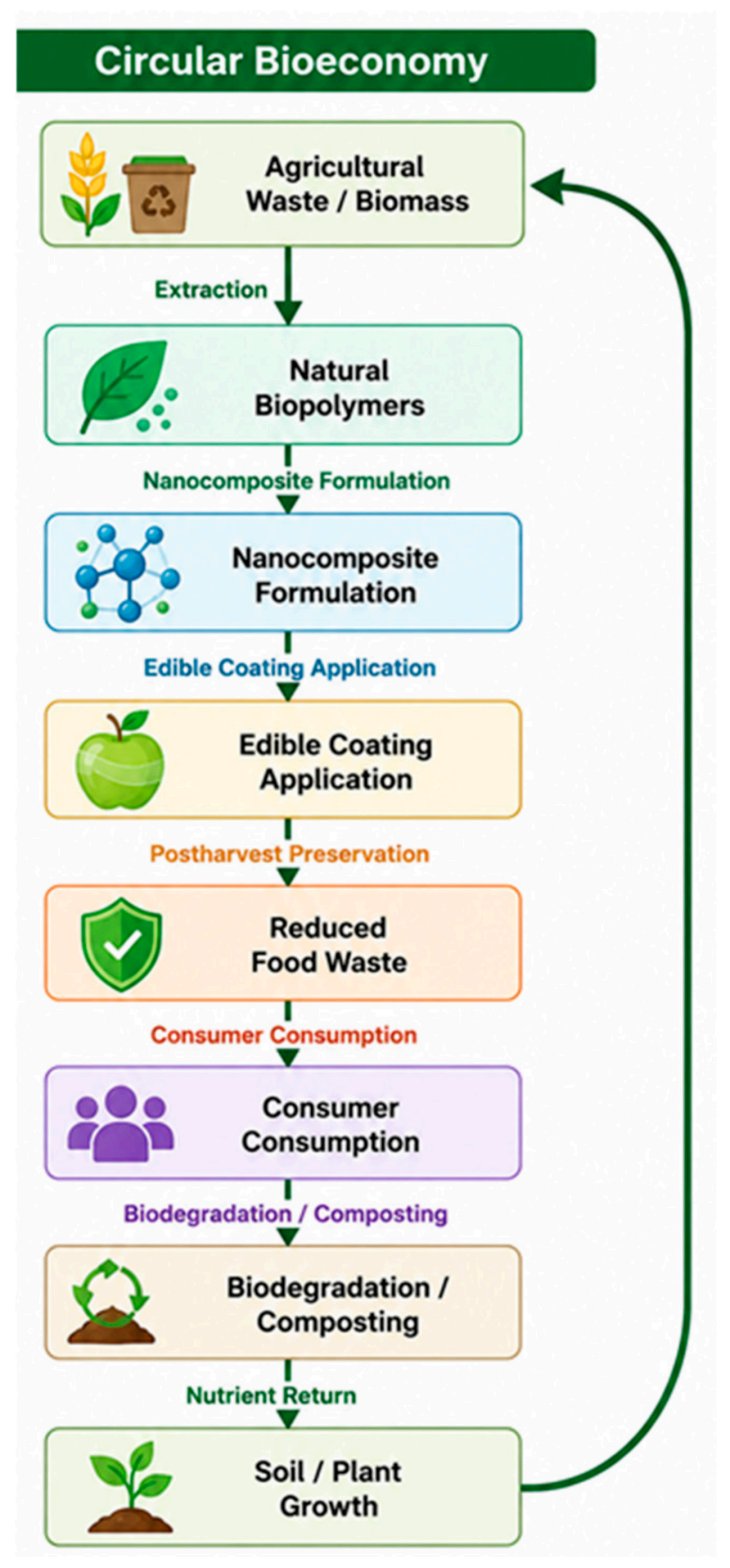
Lifecycle and sustainability of biopolymer-based edible coatings within a circular bioeconomy, from agricultural biomass to biodegradation and nutrient return.

The transition from conventional synthetic packaging to biopolymer-based edible coatings represents a conceptual shift towards more sustainable and integrated food preservation systems. While synthetic plastics provide excellent physical protection and barrier properties, their environmental persistence and limited recyclability are significant drawbacks [8]. In contrast, biopolymer-based coatings are derived from renewable biological resources and are fully biodegradable, aligning with the goals of reducing plastic waste and promoting a circular economy. This circular-bioeconomy logic is summarised in Figure 2, which traces the pathway from agricultural waste and biomass through biopolymer extraction, nanocomposite formulation and coating application, to reduced food waste, consumer use, and eventual biodegradation with nutrient return to soil. Locating the coating technology within this loop clarifies why biopolymer-based nanocomposites are treated here as a structural alternative to petroleum-derived packaging rather than as an incremental improvement on it. Moreover, the ability to incorporate active functional additives directly into the coating matrix enables more targeted and efficient delivery of antimicrobial and antioxidant protection [16]. This integrated approach not only extends the shelf life of horticultural crops but also maintains their nutritional and sensory quality, meeting the increasing consumer demand for fresh and minimally processed food.

The protective role of edible coatings is further amplified through the application of nanotechnology. The development of nanocomposite coatings involves incorporating nanoscale materials into the biopolymer matrix, leading to significant improvements in mechanical strength, gas and moisture barrier performance, and active functionalities [9]. These advancements enable the creation of more robust and effective protective layers that withstand the rigors of postharvest handling and storage. By addressing the fundamental physiological challenges through a combination of physical barrier functions and active biological protection, biopolymer-based nanocomposite edible coatings offer a comprehensive and sustainable solution for the postharvest preservation of horticultural crops [16].

## 4. Biopolymer Matrices for Edible Coatings

Biopolymer matrices form the fundamental structural component of edible coatings, providing the necessary film-forming capacity and initial barrier properties. These natural polymers are favored for their biodegradability, biocompatibility, and abundance, offering a sustainable alternative to synthetic packaging materials [17]. The selection of a specific biopolymer matrix is crucial as it dictates the coating’s mechanical strength, gas and moisture barrier efficacy, and compatibility with various functional additives. Different classes of biopolymers, including polysaccharides, proteins, and lipids, each possess unique characteristics that make them suitable for diverse applications in postharvest preservation [18]. The inherent properties of these biopolymers can be further enhanced through chemical modifications, cross-linking, or by combining them to form composite systems, thereby overcoming individual limitations and optimizing performance for specific horticultural crops [19]. The development of these biopolymer-based coatings is driven by a global shift towards sustainable food systems, aiming to reduce reliance on petroleum-based plastics and minimize environmental impact [8]. Their ability to form a semi-permeable barrier on the surface of fruits and vegetables helps to regulate physiological processes, such as respiration and transpiration, which are critical for maintaining freshness and extending shelf life. Moreover, the inherent biological activities of certain biopolymers, such as the antimicrobial properties of chitosan, provide an additional layer of protection against microbial spoilage, a major cause of postharvest losses [20].

### 4.1. Polysaccharides

Polysaccharides are a diverse group of biopolymers widely used in edible coatings for their excellent film-forming properties, high mechanical strength, and good oxygen-barrier properties. They are hydrophilic, which generally leads to poor moisture-barrier properties, but this can be mitigated by combining them with hydrophobic components or nanomaterials [21]. Commonly used polysaccharides include chitosan, starch, alginate, and cellulose derivatives. Chitosan, derived from chitin, is particularly notable for its inherent antimicrobial and antifungal properties, making it a highly effective choice for extending the shelf life of fruits and vegetables susceptible to microbial spoilage [20]. Starch, an abundant and inexpensive biopolymer, forms transparent, flexible films, while alginate, extracted from brown algae, forms strong gels in the presence of divalent cations such as calcium, providing good structural integrity. Cellulose and its derivatives, such as carboxymethyl cellulose (CMC) and hydroxypropyl methyl cellulose (HPMC), exhibit good mechanical properties and gas-barrier properties [17]. The versatility of polysaccharides enables their use across a wide range of horticultural products, with ongoing research focused on enhancing their barrier properties and integrating them with active compounds. Plant polysaccharides are also recognised for bioactive properties beyond film formation, including immunomodulatory and metabolic effects [22]. Polysaccharides from novel plant sources continue to be characterised for immune and bioactive activity, expanding the pool of candidate film-forming materials [23].

### 4.2. Proteins

Protein-based edible coatings are recognized for their superior gas barrier properties, particularly against oxygen, and their ability to form strong, flexible films. Proteins are amphiphilic, containing both hydrophilic and hydrophobic regions, which contributes to their film-forming capacity [24]. Common protein sources include whey protein, zein (from corn), soy protein, and gelatin. Whey protein is known for forming transparent films with excellent oxygen barrier properties, making it suitable for products sensitive to oxidation [25]. Zein, a prolamin protein, forms films with good mechanical strength and moderate water barrier properties. Soy protein is also widely used for its abundance, low cost, and good film-forming properties, although its mechanical properties can be improved through cross-linking. Gelatin, derived from collagen, forms strong, elastic films that can effectively reduce moisture loss and improve the appearance of coated produce [26]. The functional properties of protein films can be enhanced by denaturation, enzymatic cross-linking, or the incorporation of plasticizers and other additives. Gelatin-based composite coatings have been developed for biomedical applications requiring controlled surface bioactivity, demonstrating the versatility of gelatin as a coating base [27].

### 4.3. Lipids and Composites

Lipids are primarily incorporated into edible coatings to improve their moisture barrier properties, as their hydrophobic nature effectively retards water vapor transmission. However, lipid films alone often lack mechanical strength and are brittle, necessitating their combination with other biopolymers. Commonly used lipids include waxes (e.g., carnauba wax, beeswax), fatty acids, and essential oils. Waxes form a continuous hydrophobic layer that significantly reduces water loss from the surface of horticultural crops [15]. Essential oils, while primarily recognized for their antimicrobial and antioxidant activities, also contribute to the hydrophobic character of coatings [28]. Composite coatings, which combine two or more types of biopolymers (e.g., polysaccharide–protein, protein–lipid, or polysaccharide–lipid), are designed to leverage the synergistic benefits of each component while mitigating their individual drawbacks. For instance, a polysaccharide-lipid composite can offer both good oxygen and moisture barrier properties, along with enhanced mechanical strength, providing a more comprehensive protective solution for postharvest preservation [29]. Table 1 provides a comparative overview of biopolymers commonly used in edible coatings, highlighting their sources, key functional properties, and typical applications for the preservation of horticultural crops.

## 5. Nanomaterials in Edible Coatings: Types and Roles

The incorporation of nanomaterials into biopolymer matrices has ushered in a new era for edible coatings, transforming them into highly functional nanocomposites with enhanced properties. Nanomaterials, typically defined as materials with at least one dimension in the nanoscale (1–100 nm), exhibit unique physical, chemical, and biological characteristics due to their high surface area-to-volume ratio and quantum effects [31]. The functional contribution of a nanomaterial depends on its class. Inorganic nanoparticles act as antimicrobial agents and barrier fillers but introduce migration and toxicity considerations; organic nanofillers act primarily as mechanical and barrier reinforcements and are regarded as lower-hazard; nanoemulsions and nanocapsules function as carriers that stabilise and release hydrophobic actives, with efficacy determined by droplet stability and encapsulation efficiency [10]. The selection and dispersion of nanomaterials are critical factors influencing the overall performance and safety of the resulting nanocomposite coating. The unique properties of nanomaterials, such as their high surface area and reactivity, allow for enhanced interactions with both the biopolymer matrix and the target spoilage agents [10,32]. This leads to superior barrier performance, where the nanoparticles create a more tortuous path for gas and moisture molecules, effectively reducing their permeability through the coating. Furthermore, the ability of certain nanoparticles to generate reactive oxygen species or release antimicrobial ions provides a direct means of combating microbial growth, offering a significant advantage over traditional edible coatings [33].

### 5.1. Inorganic Nanofillers and Nanoparticles

**Figure 3:**
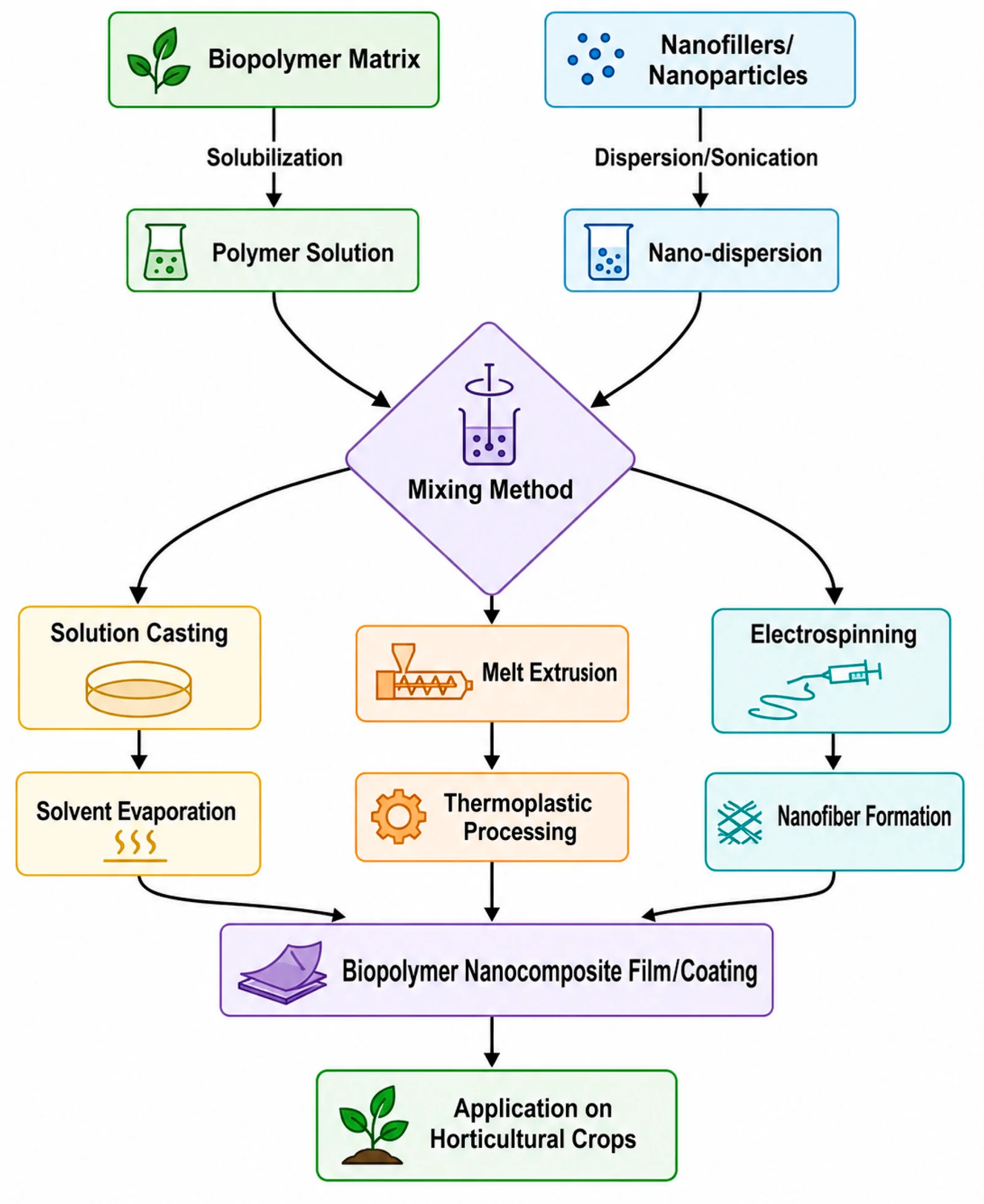
Schematic representation of the fabrication of biopolymer nanocomposites.

Inorganic nanoparticles (NPs) are among the most widely studied nanomaterials for enhancing edible coatings, primarily due to their robust antimicrobial properties and ability to improve barrier functions. Common examples include silver nanoparticles (AgNPs), zinc oxide nanoparticles (ZnONPs), titanium dioxide nanoparticles (TiO_2_NPs), and copper oxide nanoparticles (CuONPs). Silver nanoparticles are renowned for their broad-spectrum antimicrobial activity against bacteria, fungi, and viruses, acting through mechanisms such as cell membrane disruption, protein denaturation, and DNA damage [34]. Zinc oxide and titanium dioxide nanoparticles also exhibit significant antimicrobial effects, often attributed to the generation of reactive oxygen species (ROS) and interference with cellular processes [10]. CuONPs have demonstrated potent antifungal and antibacterial properties, making them valuable for combating postharvest pathogens [35]. These inorganic NPs can also enhance the mechanical strength and gas barrier properties of biopolymer films by serving as nanofillers that reinforce the polymer matrix [10]. Metal–organic frameworks represent an emerging class of antimicrobial coating material that combines metal-ion release with a tunable porous structure, and their incorporation into functional metal coatings has been reviewed for corrosion resistance and antimicrobial performance [36]. Their application in edible coatings remains exploratory, but the underlying antimicrobial mechanism controlled by metal-ion release is the same as that of the inorganic nanoparticles discussed above. Figure 3 illustrates the general process for fabricating biopolymer nanocomposites, highlighting the various methods for combining biopolymer matrices with nanofillers to produce the final coating material.

### 5.2. Bio-Based Nanofillers

Organic nanofillers, derived from natural sources, offer a biodegradable and biocompatible alternative to inorganic nanoparticles, aligning with the sustainable nature of edible coatings. Cellulose nanocrystals (CNCs), cellulose nanofibers (CNFs), and chitin whiskers (ChWs) are prominent examples. These nanofillers possess high aspect ratios, excellent mechanical properties, and strong hydrogen bonding capabilities, which enable them to significantly reinforce biopolymer matrices [37]. The incorporation of CNCs or CNFs can substantially increase the tensile strength and Young’s modulus of edible films, while also improving their oxygen barrier properties by creating a dense, ordered network within the polymer matrix [30]. Chitin whiskers, like cellulose derivatives, also enhance mechanical and barrier properties [38]. The use of organic nanofillers not only improves the physical integrity of the coatings but also maintains their natural and edible characteristics, making them highly desirable for food applications.

### 5.3. Nanoemulsions and Nanoencapsulation

Nanoemulsions and nanoencapsulation techniques are crucial for effectively incorporating hydrophobic functional additives, such as essential oils and plant extracts, into hydrophilic biopolymer matrices. Nanoemulsions are thermodynamically unstable systems consisting of nanoscale droplets (typically 20–200 nm) of one liquid dispersed in another, stabilized by surfactants [39]. Their small droplet size provides high surface area, leading to enhanced bioavailability, improved stability against oxidation, and more efficient delivery of active compounds. When incorporated into edible coatings, nanoemulsions can significantly enhance the coating’s antimicrobial and antioxidant efficacy by ensuring uniform dispersion and controlled release of active ingredients [40]. Nanoencapsulation involves entrapping active compounds within a nanoscale polymeric shell, protecting them from degradation and facilitating their controlled release [28,41]. This technique is particularly useful for volatile or sensitive compounds, ensuring their stability and prolonged activity within the coating [42]. Microencapsulation has similarly been used to preserve the bioactivity of plant-derived flavonoids during cryopreservation and storage, demonstrating that encapsulation protects polyphenolic compounds from degradation while retaining their functional activity [43]. Both nanoemulsions and nanoencapsulation contribute to the development of highly effective and stable active edible coatings, maximizing the impact of functional additives on postharvest preservation. Table 2 summarizes common nanomaterials used in edible coatings and their specific functions in fruit preservation.

## 6. Mechanisms of Action in Postharvest Preservation

Biopolymer-based nanocomposite edible coatings exert their protective effects on horticultural crops through a combination of physical, chemical, and biological mechanisms. These mechanisms help slow senescence, reduce microbial spoilage, and maintain the overall quality and nutritional value of fresh produce. The synergistic interplay between the biopolymer matrix, nanomaterials, and functional additives allows for a multi-pronged approach to postharvest preservation, addressing various factors that lead to deterioration. Understanding these mechanisms is crucial for designing and optimizing coatings tailored to the specific needs of different horticultural products [16]. The efficacy of these coatings stems from their ability to create a microenvironment around the produce that is unfavorable for spoilage. This involves passive barrier functions and active interactions with the physiological processes of the fruit or vegetable and the metabolic activities of spoilage microorganisms [47].

### 6.1. Gas Barrier Mechanisms

**Figure 4:**
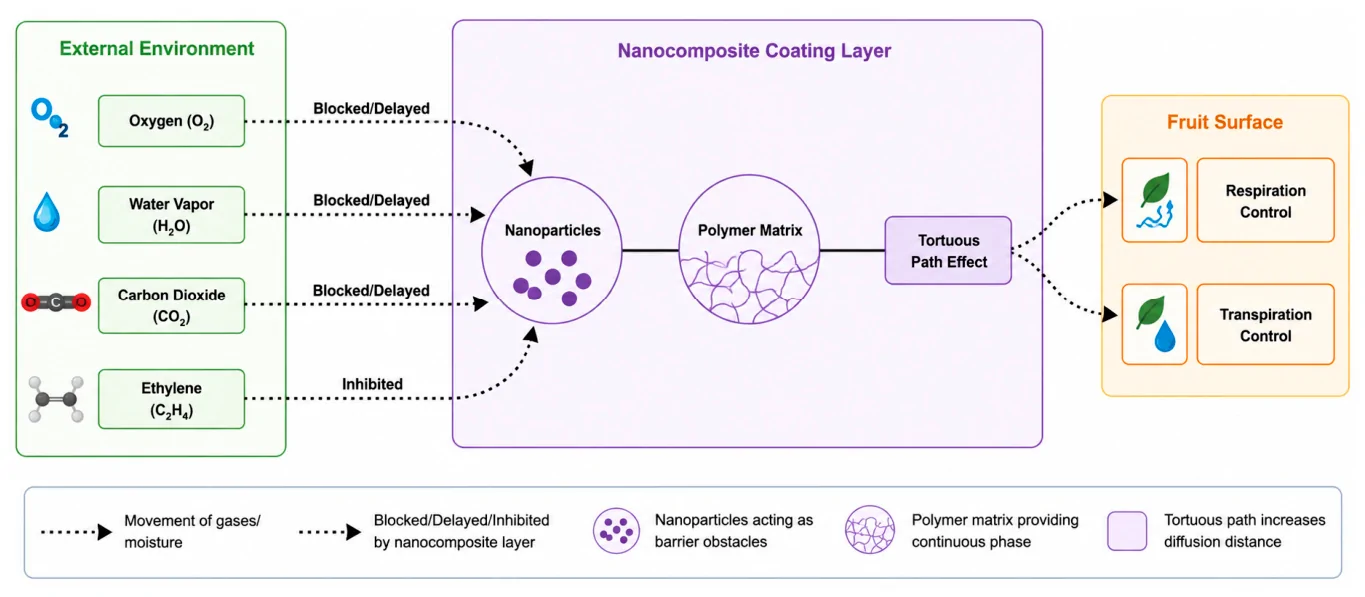
Mechanisms of gas and moisture barrier in nanocomposite coatings.

The primary functions of edible coatings are to modify the internal atmosphere surrounding the horticultural product, thereby regulating its respiration rate and delaying senescence. Coatings act as selective barriers to gases such as O_2_, CO_2_, and ethylene. Reduced oxygen permeability slows down aerobic respiration, which in turn decreases the consumption of sugars and organic acids, preserving the product’s energy reserves and extending its shelf life [5]. Simultaneously, an increased carbon dioxide concentration within the coated product can inhibit certain enzymatic activities and microbial growth. Ethylene, a plant hormone, plays a critical role in initiating and accelerating the ripening process in many fruits. Coating can either reduce the production of ethylene or act as a barrier to its diffusion, thereby delaying ripening and associated quality changes [4,48]. The incorporation of nanomaterials, such as inorganic nanoparticles or organic nanofillers, significantly enhances the gas barrier properties of biopolymer matrices. These nanoparticles increase the diffusion resistance and reduce gas exchange between the product and the external environment. This effect, coupled with the inherent barrier properties of the biopolymer, results in a more effective modified atmosphere around the produce, as illustrated in Figure 4.

### 6.2. Moisture Regulation and Transpiration Control

Water loss, primarily through transpiration, is a major cause of postharvest deterioration in horticultural crops, leading to wilting, shriveling, loss of turgor, and reduced marketability. Edible coatings form a semi-permeable barrier on the surface of the produce, effectively regulating moisture exchange with the surrounding environment [15]. While hydrophilic biopolymers like polysaccharides generally have poor water barrier properties, the incorporation of hydrophobic components, such as lipids (waxes, fatty acids) or certain nanomaterials, can significantly improve this property [21]. Lipids create a continuous hydrophobic network within the coating, reducing the water vapor transmission rate (WVTR). Nanomaterials can also contribute to moisture regulation by filling pores and increasing the density of the coating structure, thereby hindering water molecule movement. By minimizing water loss, these coatings help maintain the turgidity, freshness, and overall appearance of fruits and vegetables, extending their shelf life and preserving their economic value [49].

### 6.3. Antimicrobial and Antifungal Mechanisms

**Figure 5:**
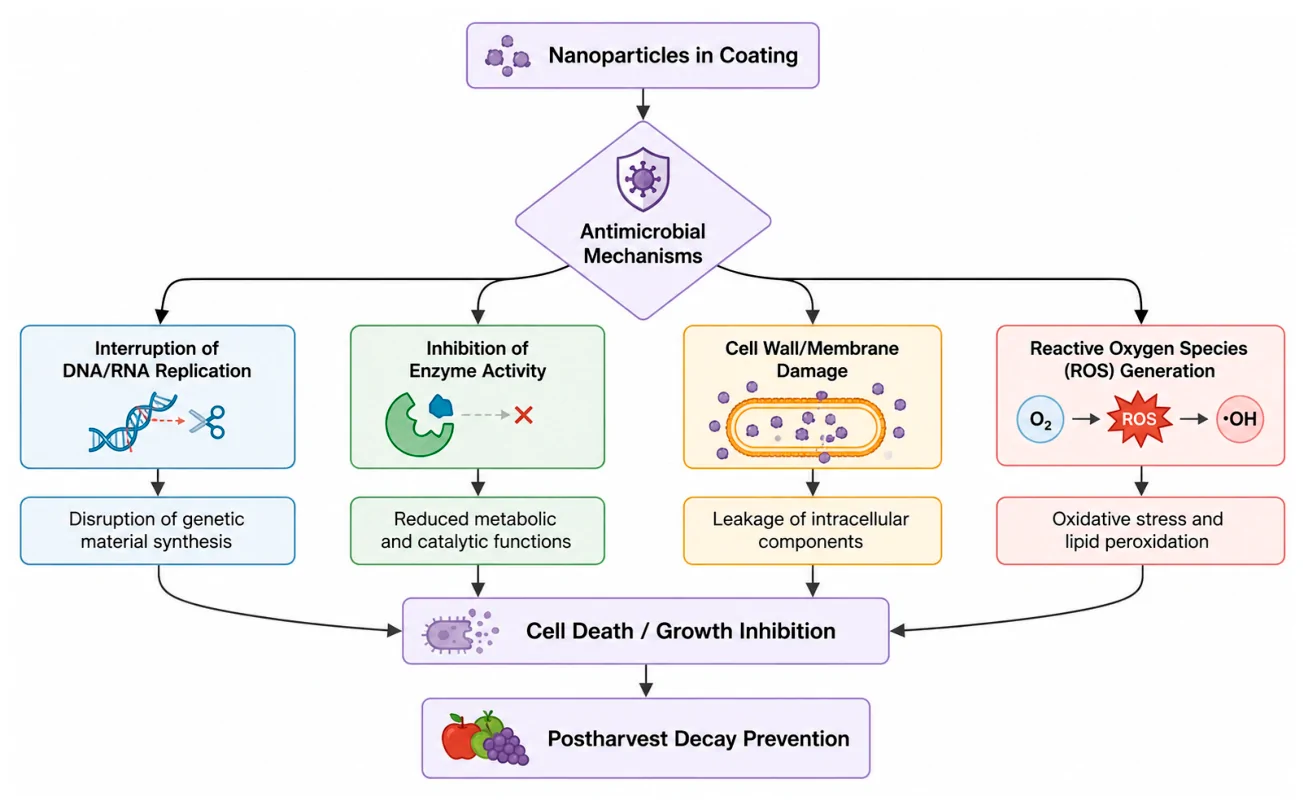
Antimicrobial action of nanoparticles on foodborne pathogens.

Microbial spoilage caused by bacteria and fungi is a leading factor in postharvest losses. Biopolymer-based nanocomposite edible coatings can actively combat these microorganisms through various mechanisms. Many biopolymers, such as chitosan, possess intrinsic antimicrobial properties due to their polycationic nature, which interacts with negatively charged microbial cell membranes, leading to membrane disruption and leakage of intracellular components [50]. The incorporation of specific nanomaterials, particularly inorganic nanoparticles such as AgNPs, ZnONPs, and CuONPs, significantly enhances the antimicrobial and antifungal efficacy of the coatings [10]. These nanoparticles release metal ions that can bind to microbial cell walls and membranes, causing structural damage and increasing permeability. They can also generate reactive oxygen species (ROS), leading to oxidative stress, damage to cellular components (proteins, lipids, DNA), and ultimately cell death [51]. Furthermore, nanoparticles can interfere with microbial metabolic pathways, enzyme activity, and DNA replication, inhibiting growth and proliferation [34]. Essential oils and plant extracts, when incorporated into the coatings, also contribute potent antimicrobial compounds that disrupt microbial cell integrity and function [52]. Figure 5 illustrates the various antimicrobial mechanisms employed by nanoparticles within edible coatings.

### 6.4. Antioxidant Defense and Enzymatic Inhibition

Oxidative stress, caused by reactive oxygen species (ROS), leads to the degradation of lipids, proteins, and pigments, resulting in browning, off-flavors, and loss of nutritional quality in horticultural crops [53]. Edible coatings can provide an antioxidant defense system, protecting the produce from oxidative damage. Many biopolymers, especially those derived from plant sources, contain natural antioxidant compounds. The incorporation of functional additives such as essential oils, plant extracts (e.g., phenolic compounds, flavonoids), and antioxidant nanoparticles (e.g., cerium oxide nanoparticles) directly enhances the antioxidant capacity of the coatings [54]. These compounds scavenge free radicals, chelate metal ions that catalyze oxidation, and inhibit the activity of pro-oxidant enzymes. By reducing oxidative stress, coatings help maintain the color, flavor, and nutritional integrity of fruits and vegetables. Additionally, coatings can act as physical barriers to oxygen, thereby limiting the substrate available for oxidative reactions. They can also modulate the activity of endogenous enzymes responsible for browning (e.g., polyphenol oxidase) and softening (e.g., pectinases), further contributing to the preservation of quality [4,5].

## 7. Functional Additives and Synergistic Effects

The efficacy of biopolymer-based nanocomposite edible coatings can be significantly enhanced through the incorporation of various functional additives. These additives are selected for their specific biological activities, such as antimicrobial, antioxidant, or anti-senescence properties, and their ability to synergistically interact with the biopolymer matrix and nanomaterials. The strategic inclusion of these compounds allows for the development of ‘active’ coatings that not only provide a physical barrier but also actively contribute to the preservation of horticultural crops. The choice of functional additives is often dictated by the specific spoilage mechanisms relevant to the target produce and the desired shelf-life extension [55]. These functional additives can be broadly categorized based on their primary mode of action, including antimicrobial agents, antioxidants, and anti-senescence compounds. Their incorporation into the coating matrix can be achieved through various methods, such as direct mixing, nanoencapsulation, or covalent grafting, each offering distinct advantages in stability, controlled release, and efficacy. The synergistic effects observed when combining multiple additives or integrating them with nanomaterials often lead to superior preservation outcomes compared to single-component systems, providing a comprehensive defense against multiple deterioration pathways [56].

### 7.1. Essential Oils and Plant Extracts

**Figure 6:**
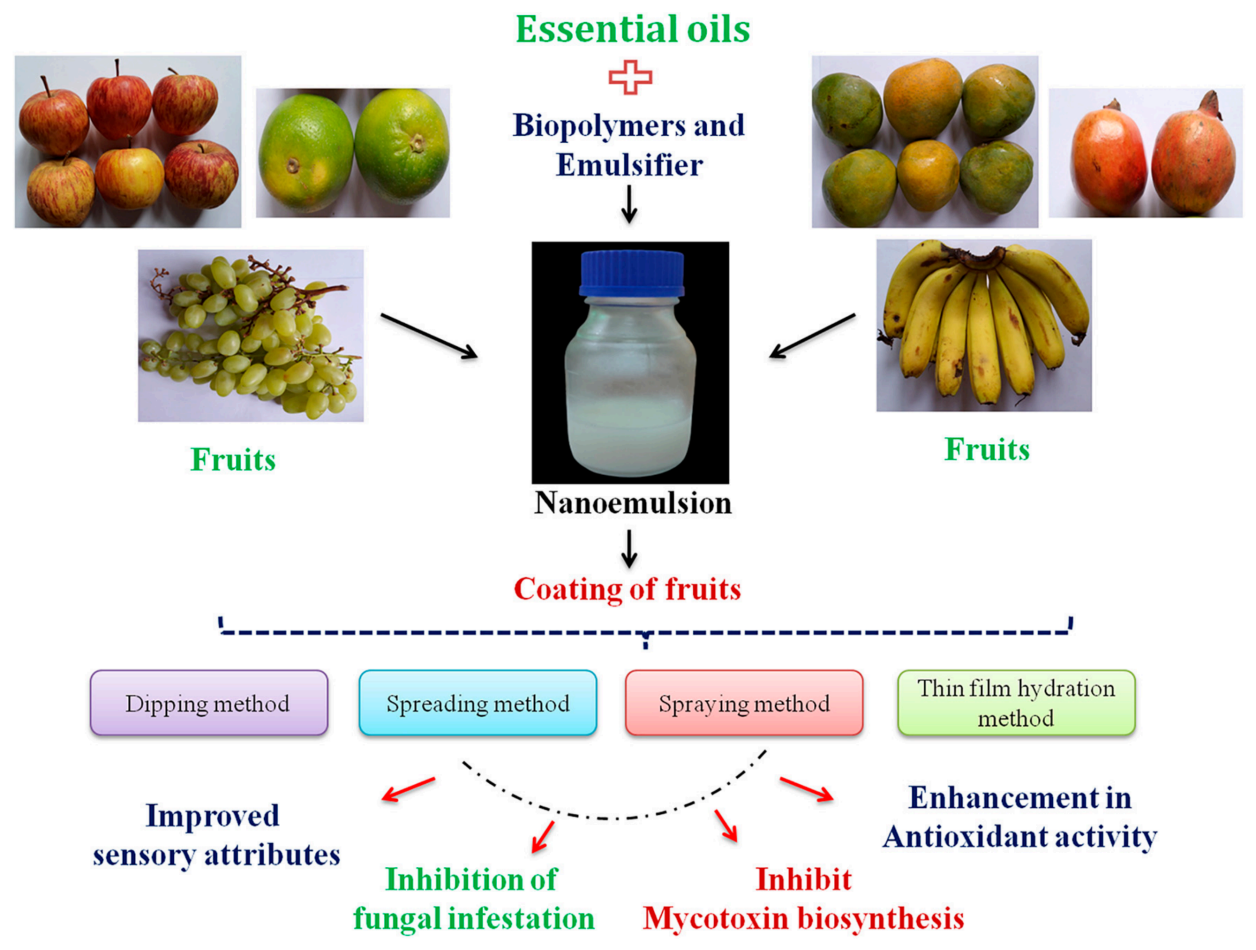
Nanoemulsion-based coating process and its effects on fruits.

Essential oils (EOs) and plant extracts are rich sources of bioactive compounds, including terpenes, terpenoids, phenolic compounds, and aldehydes, which possess potent antimicrobial, antifungal, and antioxidant properties [52]. Widely recognized for their natural origin, these additives are increasingly favored by consumers seeking ‘clean label’ products. When incorporated into edible coatings, EOs and plant extracts can inhibit the growth of a broad spectrum of postharvest pathogens, including bacteria (e.g., *Escherichia coli*, *Salmonella*) and fungi (e.g., *Botrytis cinerea*, *Penicillium digitatum*). Their mechanisms of action often involve disrupting microbial cell membranes, inhibiting enzyme activity, and interfering with cellular respiration [57]. Beyond their antimicrobial effects, many EOs and plant extracts also exhibit strong antioxidant activity, scavenging free radicals and preventing oxidative damage to the produce. Examples include oregano oil, thyme oil, cinnamon oil, rosemary extract, and green tea extract [58]. Tea polyphenols have also been engineered into nanoplatforms for the stabilised delivery of sensitive actives, illustrating the broader potential of polyphenol-based carriers beyond simple additive incorporation [59]. However, the high volatility and strong aroma of some EOs can limit their application, necessitating encapsulation techniques (e.g., nanoemulsions) to ensure controlled release and minimize sensory impact [46]. Figure 6 illustrates the nanoemulsion-based coating process and its effects on fruits, highlighting the role of essential oils.

### 7.2. Probiotics and Prebiotics

Probiotics and prebiotics are frequently mentioned as emerging coating additives, but the evidence base for their use on fresh horticultural produce is substantially weaker than for essential oils, plant extracts, or nanomaterials, and this section therefore assesses the limitations rather than the promise [11,60]. Probiotic bacteria must remain metabolically active to compete with spoilage organisms, yet edible coatings are typically dried, stored at ambient or chilled temperatures, and exposed to the low water activity and acidic surface pH of fruits [61]. Viability losses during film formation and storage are commonly reported to exceed several log cycles, and the surviving population at the point of consumption is rarely quantified. A coating that delivers an insufficient dose at the time of application, and a further reduced dose after storage, cannot be assumed to provide a competitive exclusion effect [7]. Prebiotics such as inulin, fructooligosaccharides, and galactooligosaccharides can support probiotic survival in the coating matrix, but they also alter film permeability and mechanical behaviour, and their selective effect on the target strain within a complex epiphytic microbiome has not been demonstrated on produce surfaces.

Live microorganisms added to food fall under different frameworks from conventional food additives, and the absence of harmonised international rules for probiotic-containing edible coatings on fresh produce creates uncertainty for manufacturers [49,61]. Also, surveys consistently show that consumers are more receptive to preservation technologies they perceive as natural, but live bacteria on fresh produce may be interpreted as contamination rather than as a benefit, particularly where the product is consumed raw. Given these constraints, the available evidence does not support presenting probiotics and prebiotics as established coating additives for horticultural crops. Their inclusion is better framed as an open research question requiring strain selection for produce-surface conditions, quantification of viable dose at consumption rather than at application, and consumer research on acceptability.

### 7.3. Natural Crosslinkers

Natural crosslinkers are compounds used to improve the mechanical properties, water resistance, and structural integrity of biopolymer-based edible coatings. By forming covalent or non-covalent bonds between polymer chains, crosslinkers enhance the cohesive forces within the coating matrix, leading to stronger, more durable films [19]. Common natural crosslinkers include phenolic compounds (e.g., ferulic acid, gallic acid), genipin, and transglutaminase enzyme. Phenolic compounds can interact with proteins and polysaccharides through hydrogen bonding and hydrophobic interactions, as well as through covalent bonding after oxidation, thereby improving barrier properties and mechanical strength [62]. Feruloylated oligosaccharides derived from rice bran illustrate how phenolic moieties can be conjugated to carbohydrate backbones, altering starch digestibility and interaction behaviour, and pointing to a route for designing phenolic-functionalised coating matrices [63]. Genipin, a natural crosslinker derived from gardenia fruit, is known for its low cytotoxicity and ability to form stable cross-links with proteins and chitosan [64]. Transglutaminase, an enzyme, catalyzes the formation of isopeptide bonds between protein molecules, significantly enhancing the mechanical properties and water resistance of protein-based films [65]. The use of natural crosslinkers is crucial for developing robust edible coatings that can withstand handling and storage conditions while maintaining their protective functions. Table 3 provides an overview of the synergistic effects observed when combining different functional additives within biopolymer-based nanocomposite edible coatings.

## 8. Application Methods for Horticultural Crops

The effectiveness of biopolymer-based nanocomposite edible coatings depends not only on their composition but also on the method of application. The chosen technique must ensure uniform coverage, appropriate coating thickness, and strong adhesion to the surface of the horticultural product, without causing damage or negatively impacting its sensory attributes [73]. Various application methods, ranging from conventional techniques to more advanced emerging technologies, have been developed and optimized for different types of produce and coating formulations. Each method presents distinct advantages and limitations concerning scalability, cost, and coating performance. The application method plays a crucial role in determining the structural integrity, barrier properties, and overall efficacy of the edible coating. Factors such as the viscosity of the coating solution, the surface properties of the fruit or vegetable, and the desired coating thickness must be carefully considered when selecting a method [74]. Proper application ensures that the coating forms a continuous, uniform layer, maximizing its protective functions while minimizing potential negative impacts on the produce, such as stomatal clogging or uneven drying.

### 8.1. Conventional Methods

**Figure 7:**
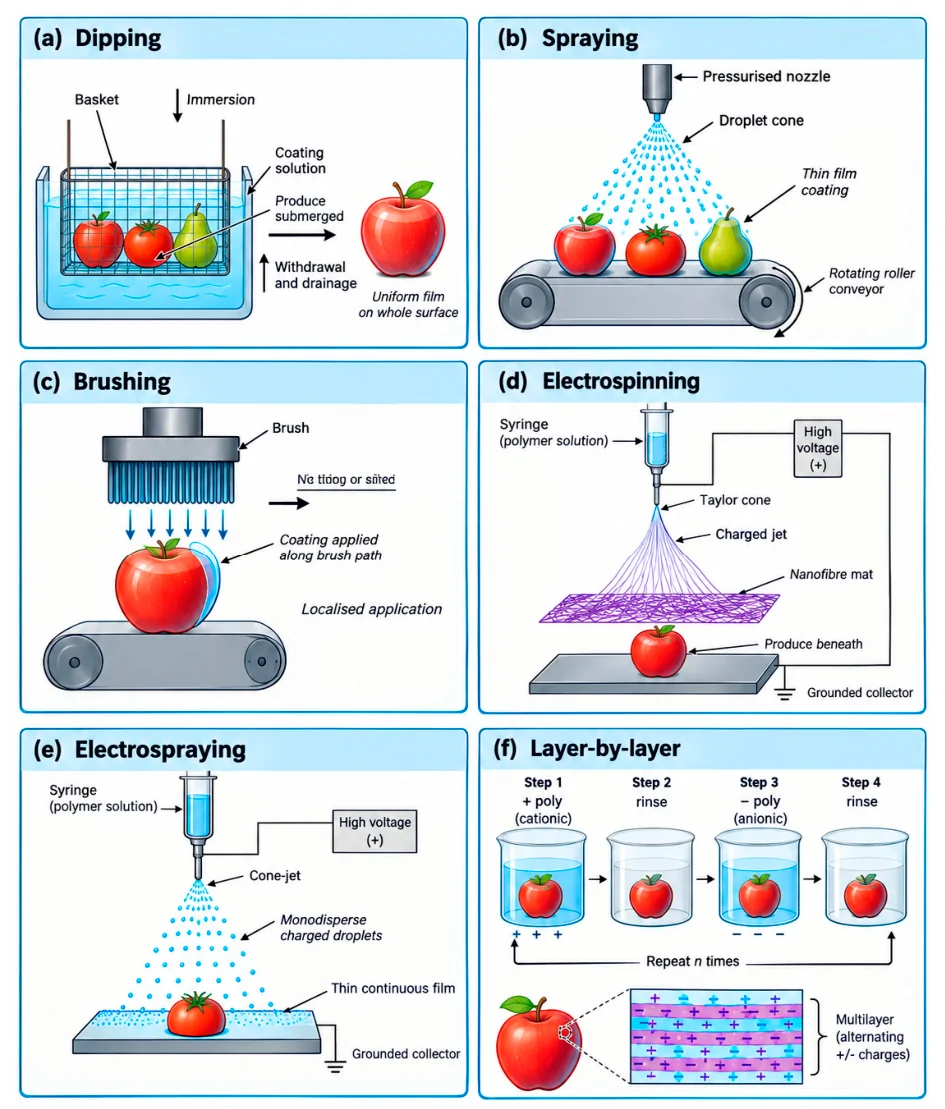
Application methods for edible coatings on horticultural produce: (**a**) dipping, (**b**) spraying, (**c**) brushing, (**d**) electrospinning, (**e**) electrospraying, and (**f**) layer-by-layer assembly.

Conventional application methods are widely adopted in the food industry due to their simplicity, cost-effectiveness, and ease of large-scale implementation. These methods include dipping, spraying, and brushing. Dipping involves immersing the horticultural product in a coating solution for a specific duration, allowing the coating to adhere to its surface. This method is straightforward and ensures complete coverage, making it suitable for irregularly shaped fruits and vegetables. However, it can lead to excessive coating thickness and potential contamination of the dipping solution over time [75]. Spraying involves atomizing the coating solution and applying it as a fine mist onto the produce. This technique offers better control over coating thickness and uniformity, reduces material waste, and is easily adaptable to automated production lines [73,76]. It is particularly effective for delicate fruits that may be damaged by dipping. Brushing involves manually or mechanically applying the coating solution onto the surface using brushes. While simple, this method is generally less uniform and more labor-intensive, making it better suited to small-scale operations or specific applications that require precise control over localized coating. Figure 7 illustrates the conventional and emerging methods discussed in this section as applied to whole produce, indicating how each technique deposits the coating and the surface layer it produces.

### 8.2. Emerging Technologies

**Figure 8:**
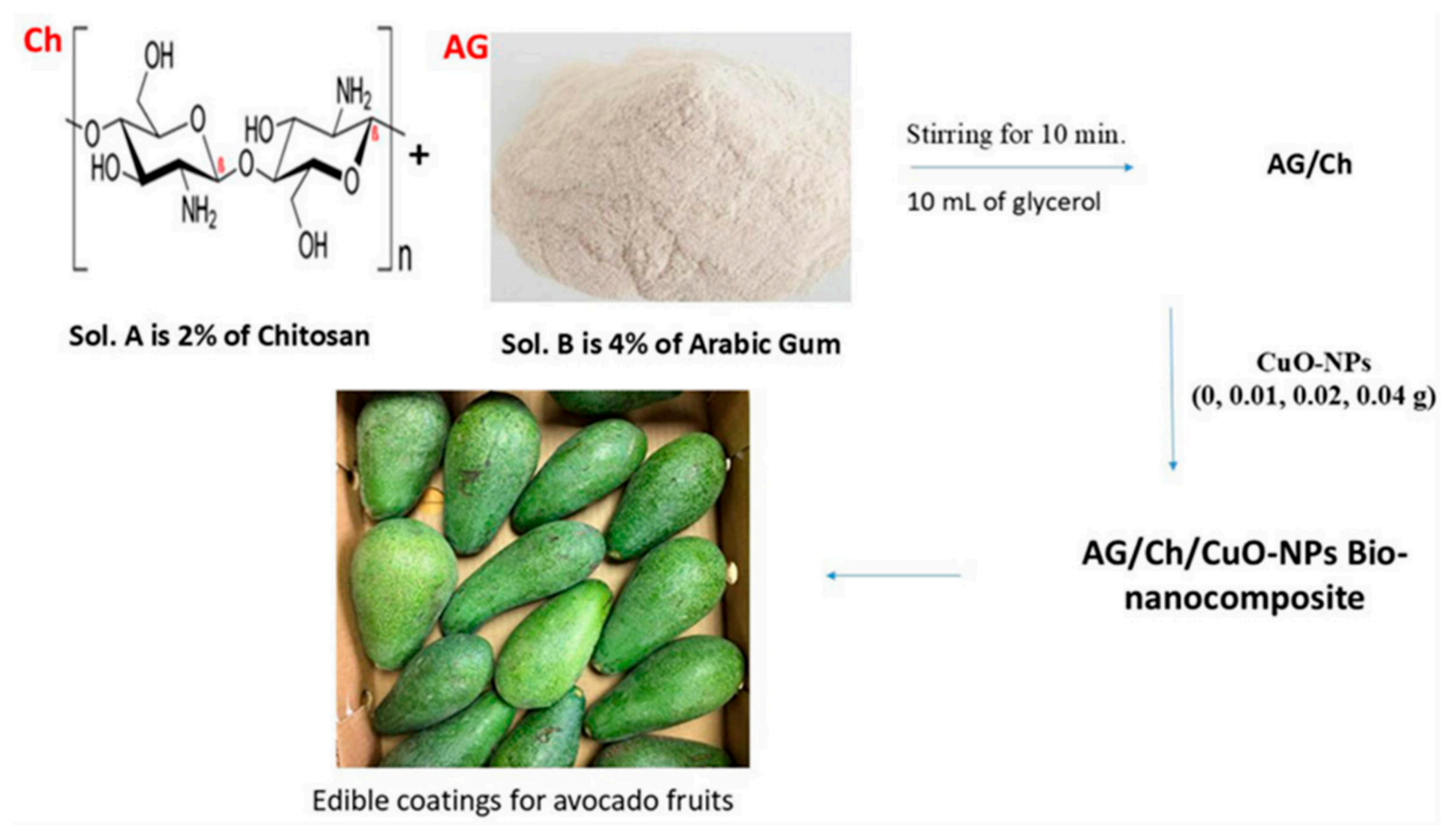
Synthesis of AG/Ch/CuO-NPs bio-nanocomposite [45]. Adapted from Abd El-Aziz et al. [45] with permission from Springer Nature, copyright 2026.

Emerging technologies offer advanced solutions for applying edible coatings, enabling greater precision, control over coating structure, and enhanced functionality. These methods often leverage principles of nanotechnology to create highly uniform and effective protective layers [77]. Electrospinning is a technique that uses an electric field to draw a charged polymer solution into ultrafine fibers, which are then deposited onto the surface of the produce to form a nanofibrous coating [78]. Electrospun nanofibre systems have been developed into functional nano-skins in adjacent biomedical fields, where in situ growth of lubricated coatings on fibre surfaces has been used to prevent post-operative adhesion [79]; the same interfacial engineering principles are transferable to produce surfaces where adhesion and friction during handling contribute to surface damage. This method enables the creation of highly porous coatings with a large surface area, which can be beneficial for the controlled release of active compounds and enhanced gas exchange. Electrospraying, like electrospinning, involves atomizing a liquid into fine droplets using an electric field, resulting in a highly uniform and thin coating layer [80]. This technique is particularly useful for applying nanoemulsions or solutions containing nanoparticles, ensuring their even distribution within the coating. Layer-by-Layer (LbL) assembly is a versatile technique that involves sequentially depositing alternating layers of oppositely charged polyelectrolytes (biopolymers) onto the surface of the produce [81]. This method allows for precise control over coating thickness, composition, and architecture, enabling the creation of multi-functional coatings with tailored barrier and active properties. These emerging technologies, while offering superior performance, often require specialized equipment and may pose challenges to scalability and cost-effectiveness in large-scale industrial applications [6]. Figure 8 illustrates the synthesis of a bio-nanocomposite and its application [45].

### 8.3. Nanoparticle-Based Coating Systems

Conventional application methods were developed for homogeneous biopolymer solutions; when the formulation contains nanoparticles, nanoemulsions or nanoencapsulated actives, the method must additionally preserve colloidal stability, prevent agglomeration and deliver the nanoscale phase uniformly [82,83]. Dipping is most widely used because it suits irregular produce, but nanoparticles settle in the bath, producing inconsistent doses across batches; agitation and replenishment are therefore required. Spraying and electrospraying give better control over deposited nanomaterial loading, and electrospraying is particularly suited to nanoparticle dispersions because the electric field limits droplet coalescence, although formulation viscosity and conductivity constrain the process. Electrospinning generates nanostructure in situ, protecting sensitive payloads within fibres and moderating release, but the porous deposit offers a weaker moisture barrier. Layer-by-layer assembly provides the greatest architectural control, enabling nanoparticle-polyelectrolyte multilayers with tuned release, yet remains the least industrially mature. Two safety considerations are method-specific: atomisation can generate airborne nanoparticles requiring containment, and improved dispersion increases the surface area available for migration, linking method selection to the migration testing in Section 10.1 [82,83]. Table 4 provides a comparative analysis of various application methods for edible coatings, highlighting their advantages and disadvantages.

## 9. Impact on Physicochemical and Sensory Quality

Biopolymer-based nanocomposite edible coatings are designed not only to extend the shelf life of horticultural crops but also to maintain or even enhance their physicochemical and sensory quality attributes. The effectiveness of these coatings is often evaluated by their ability to maintain key quality parameters critical to consumer acceptance and nutritional value. A well-formulated and properly applied coating should minimize undesirable changes while preserving the natural characteristics of the produce [75]. The preservation of quality attributes is paramount for consumer satisfaction and market value. Coatings achieve this by mitigating various postharvest deterioration processes, including moisture loss, oxidative damage, and microbial spoilage. By maintaining turgidity, color, flavor, and nutritional content, these coatings ensure that horticultural products retain their fresh-like qualities for longer periods, thereby reducing waste and enhancing consumer appeal [54]. The careful balance between effective preservation and minimal alteration of natural characteristics is a key objective in the development of these advanced coating systems [16].

### 9.1. Weight Loss and Firmness

Weight loss in horticultural crops after harvest is primarily due to evaporation of moisture (transpiration) and respiration. Edible coatings, by forming a semi-permeable barrier, significantly reduce water loss, thereby minimizing weight loss and maintaining the turgidity of the produce [47]. This is particularly important for fruits and vegetables with high water content. The preservation of firmness is closely linked to water content and the integrity of cell walls [84]. Coatings can help maintain firmness by reducing water loss and by slowing down enzymatic degradation of cell wall components (e.g., pectinases) [16]. Nanocomposite coatings, with their enhanced barrier properties and mechanical strength, are particularly effective in preserving both weight and firmness, contributing to a longer shelf life and better marketability [10].

### 9.2. Color and Appearance

Color is a critical visual attribute that significantly influences consumer perception and acceptance of horticultural crops. Changes in color during postharvest storage are often associated with ripening, senescence, or enzymatic browning [85]. Edible coatings can help maintain the natural color of fruits and vegetables by regulating gas exchange (e.g., reducing oxygen availability to inhibit browning enzymes such as polyphenol oxidase), minimizing moisture loss (which can cause dullness), and incorporating antioxidant additives that prevent pigment degradation [29]. The transparency of the coating is also important to ensure that the natural appearance of the produce is not obscured. Nanocomposite coatings can be engineered to be highly transparent while providing superior protection, thus preserving the aesthetic appeal of the coated products [5].

### 9.3. Nutritional Quality (Vitamins, Phenolics)

Horticultural crops are valued for their rich nutritional content, including vitamins, minerals, and health-promoting phytochemicals such as phenolic compounds and carotenoids. These compounds are susceptible to degradation during postharvest storage due to oxidation, enzymatic activity, and other metabolic processes [86]. Edible coatings, especially those enriched with antioxidant nanomaterials and plant extracts, can significantly preserve the nutritional quality of coated produce [73]. By reducing oxidative stress and slowing metabolic activity, coatings help maintain levels of sensitive vitamins and phenolic compounds [61]. This preservation of nutritional value is a key benefit of using active edible coatings, contributing to the health benefits derived from consuming fresh fruits and vegetables [55].

### 9.4. Sensory Acceptance

Ultimately, the success of any postharvest preservation technology depends on consumer acceptance, which is largely driven by sensory attributes such as taste, aroma, texture, and overall mouthfeel [87]. Edible coatings must not negatively alter these characteristics. While some functional additives, like essential oils, can impart strong aromas, careful formulation and encapsulation techniques can mitigate undesirable sensory impacts [50]. Ideally, coatings should be tasteless, odorless, and imperceptible to the consumer, allowing the natural flavor and aroma of the produce to prevail. Studies on consumer perception often evaluate the impact of coatings on texture (e.g., crispness, juiciness), flavor intensity, and overall liking. The goal is to develop coatings that enhance preservation without compromising the sensory experience, thereby ensuring consumer preference and market success [88]. Table 5 summarizes recent studies (2023–2026) on the application of biopolymer-based nanocomposite edible coatings to specific horticultural crops, highlighting key findings and observed benefits.

### 9.5. Quantitative Performance Metrics and Reporting Standards

Coating performance is routinely measured but rarely reported in a form permitting cross-study comparison. Water vapor transmission rates span more than an order of magnitude with matrix, hydrophobic content and test conditions; oxygen and CO_2_ transmission likewise depend on formulation, and the CO_2_/O_2_ selectivity ratio [90], the parameter that most directly predicts internal atmosphere modification is seldom reported [96,97]. Thickness ranged from 0.08 to 0.19 mm but is inconsistently reported as dry or wet, introducing systematic error; contact angles ranged from hydrophilic to 106.4°, with the increase attributable to surface enrichment of phosphorylated zein, although measurement time is rarely specified. Tensile strength rose from 30.8 to 37.5 MPa as reinforcement increased while elongation fell from 10.0% to 7.8%, illustrating the strength–ductility trade-off [98]. Antimicrobial activity is reported as log reduction, inhibition zone or MIC, preventing comparison; where log reduction is given, values typically fall between 1 and 4 log CFU g^−1^ and are meaningful only when inoculum level, temperature and matrix are specified. Antioxidant activity depends on assay (DPPH, ABTS, FRAP, ORAC) and expression basis. We therefore recommend minimum reporting requirements covering OTR, WVTR, thickness, contact angle, mechanical properties, antimicrobial log reduction and antioxidant assay, so that findings become cumulative. Table 6 consolidates these metrics with units, test conditions and sources.

## 10. Safety, Regulatory, and Toxicological Perspectives

The increasing use of biopolymer-based nanocomposite edible coatings in the food industry necessitates a thorough understanding of their safety, regulatory compliance, and potential toxicological implications. While biopolymers are generally recognized as safe (GRAS) and derived from natural sources, the incorporation of nanomaterials introduces new considerations regarding their interaction with food matrices, potential migration into the food, and their effects on human health and the environment. Ensuring the safety of these advanced coatings is paramount for consumer trust and successful commercialization [101]. The assessment of safety extends beyond the individual components to consider the potential interactions within the nanocomposite system and their behavior in various food environments. This includes evaluating the stability of nanoparticles within the coating, their potential for release and migration into the food, and their fate upon ingestion. Comprehensive toxicological studies, including in vitro and in vivo assessments, are essential for establishing the safety profiles of these materials and informing regulatory decisions. Harmonized international regulations and clear guidelines are crucial for fostering innovation while safeguarding public health [102].

### 10.1. Migration of Nanoparticles

One of the primary safety concerns associated with nanocomposite edible coatings is the potential migration of nanoparticles from the coating into the food product. The small size of nanoparticles allows for greater mobility, and factors such as coating composition, food matrix characteristics, storage conditions (temperature, pH), and the type of nanomaterial can influence migration rates. Studies are conducted to quantify the extent of nanoparticle migration and assess whether the levels are within acceptable safety limits. While some studies report minimal or no migration, others indicate that certain nanoparticles, particularly those with high solubility or small size, may migrate into the food [60]. The potential for migration necessitates rigorous testing and risk assessment to ensure that consumers are not exposed to harmful levels of nanoparticles [101]. Migration behaviour differs by class and cannot be generalised. For silver nanoparticles, EFSA found that particles remained embedded in the food-contact polymer while silver migrated in soluble ionic form at up to 6 μg/kg food, below the 50 μg/kg group restriction; the distinction between particulate migration and ionic release is therefore decisive and is frequently conflated in the coating literature [103]. For TiO_2_, EFSA [103] concluded that E171 can no longer be considered safe because a genotoxicity concern could not be excluded, a conclusion not shared by all international bodies. For organic nanofillers, translocation is constrained by the digestible or inert nature of cellulose and chitin, and the residual concern is surface modification and processing residues. For nanoemulsions, release is governed by droplet stability and encapsulation efficiency, so the relevant exposure is to the encapsulated active rather than to a persistent nanomaterial [42]. Migration measured at a single time point cannot characterize a coating that releases over weeks; time-series migration testing is therefore required, and is discussed in Section 10.5.

### 10.2. Toxicity of Nanoparticles

Nanoparticle toxicity depends on size, surface charge, dissolution, aggregation and food-matrix context, not chemical composition alone, so bulk-phase data cannot be extrapolated to the nanoscale. For edible coatings the exposure route is oral, and a food-specific adverse outcome pathway links endocytic lysosomal uptake to lysosomal disruption, mitochondrial dysfunction and intestinal barrier impairment, with supporting evidence for silver, copper oxide and silicon dioxide nanomaterials but not nanocellulose [104]. Silver nanoparticles are cytotoxic and genotoxic in vitro through oxidative stress and DNA damage, though migration from chitosan coatings has remained within safety limits [105]. Zinc oxide nanoparticles cause hepatic and renal histopathological changes in rodents, partly attributable to dissolved Zn^2+^ rather than nanoparticle-specific effects. Titanium dioxide is the most contested: EFSA [103] could not exclude a genotoxicity concern for E171, with subsequent evidence of immunomodulatory and autophagy-related effects, although food-matrix aggregation can attenuate reactivity. Organic nanofillers such as cellulose nanocrystals show no toxicity-related events in reviewed studies and represent a lower-hazard category. Two limitations constrain interpretation: most studies use pristine particles rather than coating-derived ones, and mechanistic endpoints are rarely measured in food-relevant models [103,104,105].

The environmental dimension has received less attention than human health. Silver nanoparticles show well-documented aquatic toxicity through ionic release; ZnO and CuO dissolve partly in environmental media, so exposure may be predominantly to metal ions; TiO_2_ is persistent; and cellulose nanofillers are expected to biodegrade, although the fate of surface-modified variants is unknown [60]. Long-term assessment is constrained by the absence of chronic dietary studies using coating-derived particles under realistic storage and consumption scenarios, as most toxicological evidence derives from pristine laboratory materials rather than from particles aged in a biopolymer matrix [101]. Safety assessment should therefore be organised by nanomaterial class, distinguish particulate from dissolved exposure, and prioritise application-relevant migration and chronic exposure data over additional acute in vitro studies.

### 10.3. Regulatory Status (FDA, EFSA)

The regulatory landscape for nanomaterials in food contact applications, including edible coatings, is still evolving and varies across regions. Regulatory bodies such as the U.S. Food and Drug Administration (FDA) and the European Food Safety Authority (EFSA) are actively developing guidelines and frameworks to assess the safety of nanotechnology in food. In the United States, substances used in food contact materials must be authorized by the FDA, either through a food additive petition or as GRAS. For nanomaterials, a premarket notification process is often required, where manufacturers must demonstrate that the substance is safe under its intended conditions of use. In the European Union, novel foods and food additives, including those containing nanomaterials, are subject to pre-market authorization based on a comprehensive safety assessment by EFSA. The lack of harmonized global regulations and specific guidelines for all types of nanomaterials poses challenges for manufacturers and can hinder the widespread adoption of nanocomposite coatings. Compliance with existing and emerging regulations is crucial for market entry and consumer confidence.

### 10.4. Consumer Perception

Consumer perception plays a significant role in the acceptance and market success of novel food technologies, including edible coatings containing nanomaterials. While consumers generally favor natural and sustainable preservation methods, concerns about nanotechnology in food often arise from a lack of understanding, perceived risks, and ethical considerations [77,106]. Transparency in labeling, clear communication about the benefits and safety of nanocomposite coatings, and public education initiatives are essential to build consumer trust. Studies on consumer attitudes indicate a willingness to accept nanotechnology in food if clear benefits (e.g., extended shelf life, reduced food waste) are demonstrated and safety is assured by regulatory bodies [107]. Addressing consumer concerns through scientific evidence and effective communication strategies is vital for the successful integration of these innovative coatings into the food supply chain. Table 7 provides an overview of the regulatory status of common nanocomposite ingredients in key regions.

### 10.5. Time-Dependent Coating Degradation and Crop Update

A coating is not a static barrier, so performance and safety measured on day 0 are not those that apply on day 30. Over storage the biopolymer matrix hydrates, swells and depolymerises, which plasticises polysaccharide films, increases gas permeability and progressively weakens the modified-atmosphere effect, while protein films are vulnerable to enzymatic and microbial proteolysis; barrier performance therefore decays non-linearly and shelf-life claims based on initial OTR or WVTR overstate protection. Active agents are released with time: essential oils and phenolics volatilise within days unless encapsulated, nanoencapsulation and layer-by-layer assembly extend but do not eliminate the initial burst, and nanoparticle release is governed by dissolution, desorption and matrix erosion, with silver migrating predominantly in ionic rather than particulate form, so release continuing over weeks increases the cumulative dose to the produce surface and the consumer and renders single time-point migration tests insufficient. Released nanoparticles and metal ions can also be taken up through stomata, lenticels and cuticular pathways, where they may either deliver the intended antimicrobial effect or accumulate in edible tissues and enter the food chain, with translocation governed by particle size, surface charge and cuticle condition; the crop is thus a compartment in the exposure pathway rather than a passive substrate, a factor rarely accounted for in coating studies. Coating evaluation should therefore include time-series measurements of barrier performance, active release, nanoparticle migration and, where feasible, tissue uptake at multiple storage time points rather than only at application.

## 11. Comparative Analysis of Recent Review Papers

To contextualize the present work and highlight its unique contributions, a comparative analysis of recent review papers focusing on biopolymer-based nanocomposite edible coatings for postharvest preservation is essential. This section aims to identify gaps in existing literature, underscore the novelty of this review, and provide readers with a clear understanding of how this manuscript advances the current knowledge base. The comparison will be based on several key features, including the scope, depth of analysis, specific focus areas, and the recency of the literature covered. This comparative analysis demonstrates the comprehensive nature and distinct focus of the current review. By systematically evaluating other prominent works in the field, we can identify areas where this manuscript provides novel insights, integrates a broader range of topics, or offers a more in-depth discussion on specific aspects such as safety and regulatory perspectives. This approach ensures that the present review contributes meaningfully to the existing body of knowledge, providing a valuable resource for researchers and practitioners alike. Table 8 compares this review with four other prominent review papers published in recent years. None of the compared reviews takes nanoparticle-containing coatings as its primary analytical object; each treats nanomaterials as one component of a broader coating discussion. This is the specific gap the present review addresses, and it is why the safety, migration and toxicity sections are developed at class-specific rather than general level.

## 12. Challenges and Future Directions

Despite significant advancements in biopolymer-based nanocomposite edible coatings for postharvest preservation, several challenges remain that must be addressed to facilitate their widespread industrial adoption and maximize their potential. Overcoming these hurdles will pave the way for more sustainable and effective solutions in the horticultural supply chain. The transition from laboratory-scale research to industrial application presents a complex set of challenges, including economic viability, regulatory compliance, and consumer acceptance [47]. While the scientific community has made significant strides in developing highly effective coating formulations, their practical implementation requires careful consideration of factors such as cost-effectiveness, production scalability, and the ability to integrate these technologies into existing postharvest handling systems [30]. Addressing these challenges will require interdisciplinary collaboration among scientists, engineers, policymakers, and industry stakeholders to ensure that the benefits of these innovative coatings can be realized globally.

### 12.1. Techno-Economic Structure of Coating Production and Application

The economics of nanocomposite coatings are governed by four cost centres whose relative weights differ from those of conventional packaging: raw materials, nanomaterial synthesis, film or dispersion formation, and application. Raw biopolymer is rarely the dominant cost. Chitosan, starch, alginate and whey protein are commodity or by-product streams, and cellulose nanofibrils can be recovered from agricultural residues such as maize husk, which displaces rather than adds to feedstock cost [37]. The dominant material cost is typically the nanomaterial or the encapsulated active: laboratory-scale synthesis of AgNPs, ZnONPs or CuONPs involves precursor salts, reducing agents, purification and drying steps whose cost per kilogram is one to two orders of magnitude above that of the biopolymer matrix, and this ratio does not improve proportionally with scale because purification and quality control remain batch operations.

Formation and application dominate energy demand. Solution casting requires solvent evaporation over hours at moderate temperature and is the least energy-efficient route per unit of film; melt extrusion is more energy-intensive per hour but continuous and therefore lower per unit output; electrospinning and electrospraying consume less thermal energy but operate at low throughput, typically millilitres per hour per nozzle, so energy per kilogram of deposited material remains high [111]. For coatings applied directly to produce, the drying step is the principal energy sink, and its cost scales with coating water content rather than with nanomaterial loading. This has a practical consequence: reformulating to reduce solids content to improve spreadability increases the energy required to remove the added water, a trade-off that is not captured when coating performance is reported per unit of active compound.

Equipment investment is lowest for dipping and spraying, which use vessels, pumps and nozzles already present in packinghouses, and highest for electrospinning, electrospraying and layer-by-layer assembly, which require high-voltage supplies, controlled-atmosphere enclosures and, for layer-by-layer, multiple sequential immersion stations with rinsing and drying between steps [111]. The investment asymmetry explains the adoption pattern better than cost per kilogram of coating material: dipping and spraying can be trialled with marginal capital expenditure, whereas the emerging methods require a dedicated line and therefore a demonstrated return before commitment. Table 9 compares the principal coating formation and application routes on the criteria that determine industrial adoption rather than laboratory performance: capital intensity, throughput, dominant energy demand, nanomaterial utilisation efficiency, and the specific barrier that limits scale-up. The comparison shows that the routes with the best barrier and controlled-release performance (electrospinning, electrospraying, layer-by-layer) are also those with the highest capital intensity and lowest throughput, while dipping and spraying—which are already available in most packinghouses—offer the weakest control over nanomaterial dose. No single route is optimal across all criteria, which is why method selection must be matched to the value of the crop and the loss pathway being addressed [8,111].

### 12.2. Return on Investment and Scale-Up Barriers

The return on investment calculation for a coating is determined by the value of avoided loss rather than by the cost of the coating itself. For a commodity where postharvest loss is high and unit value is moderate, a coating that reduces loss by a few percentage points can repay its cost within a single season. For a high-value crop with low baseline loss, the same coating cannot, which is why the economic case is commodity-specific and cannot be generalised from laboratory shelf-life data. Three factors make the calculation harder than it appears. First, shelf-life extension only creates value if the additional storage time can be sold; in supply chains already operating at capacity, extended shelf life reduces waste but does not increase revenue. Second, coating cost accrues per unit of produce treated, while the benefit accrues per unit of loss avoided, so the ratio worsens as coating efficacy declines late in storage. Third, the cost of regulatory compliance, including migration testing and any required notification, is fixed and therefore disproportionately burdens small producers [8,75]. Four scale-up barriers follow from this structure. Batch consistency of nanomaterial dispersion degrades with volume, because mixing intensity and residence time vary across larger vessels, and the resulting aggregation reduces both barrier performance and antimicrobial efficacy. Quality control for nanoparticle size distribution and loading requires instrumentation that packinghouses do not possess. Worker exposure during atomisation of nanoparticle-containing formulations requires containment that is not part of standard line design.

### 12.3. Smart and Active Packaging Integration

The future of postharvest preservation lies in the integration of edible coatings with smart and active packaging technologies. Smart packaging incorporates indicators or sensors that provide real-time information about the quality and safety of the product (e.g., freshness indicators, pathogen detection). Active packaging, on the other hand, actively interacts with the food or its environment to extend shelf life (e.g., oxygen scavengers, ethylene absorbers). Combining the protective barrier of edible coatings with the functionalities of smart and active components can create highly sophisticated and responsive preservation systems. Challenges include integrating sensors and active compounds seamlessly into the coating matrix without compromising their function or the coating’s integrity. Developing coatings that can respond to environmental changes (e.g., pH, temperature) by releasing active compounds in a controlled manner is another promising area. This integration will lead to next-generation packaging solutions that not only preserve horticultural crops but also monitor and adapt to changing conditions.

## 13. Conclusions

Biopolymer-based nanocomposite edible coatings represent a transformative approach to postharvest preservation of horticultural crops, offering a sustainable and effective alternative to conventional methods. This comprehensive review highlights the intricate mechanisms through which these advanced coatings extend shelf life, including their roles in gas barrier modification, moisture regulation, and providing potent antimicrobial and antioxidant defense. The synergistic integration of diverse biopolymer matrices (polysaccharides, proteins, lipids) with various nanomaterials (inorganic nanoparticles, organic nanofillers, nanoemulsions) and functional additives (essential oils, plant extracts, natural crosslinkers) enables the creation of highly tailored, multifunctional protective layers. We have explored both conventional and emerging application methods, emphasizing the importance of uniform coverage and optimal coating performance. Furthermore, critical safety, regulatory, and consumer perception aspects have been addressed, underscoring the need for rigorous testing and transparent communication to ensure widespread acceptance. While challenges related to industrial scalability, cost-effectiveness, and full integration with smart packaging technologies persist, continuous advancements in materials science and nanotechnology promise a future in which these innovative coatings play a pivotal role in reducing global food waste, enhancing food security, and promoting environmental sustainability. Ongoing research and development in this field are crucial to unlocking the full potential of biopolymer-based nanocomposite edible coatings, thereby leading to a more resilient and sustainable food system for future generations.

## Figures and Tables

**Table 1:** Properties of various biopolymers used in nanocomposite coatings.

Biopolymer Type	Source	Key Functional Properties	Advantages	Disadvantages	Typical Applications	References
**Polysaccharides**						
Chitosan	Crustacean shells, Fungi	Antimicrobial, Antifungal, Film-forming, Gas barrier	Biodegradable, Biocompatible, Non-toxic, Inherent antimicrobial activity	Poor water barrier, pH-sensitive solubility	Fruits, Vegetables, Meat, Seafood	[20,21]
Starch	Corn, Potato, Rice, Wheat	Film-forming, Gas barrier, Thickening agent	Abundant, Inexpensive, Biodegradable, Transparent films	Poor water barrier, Low mechanical strength, Retrogradation	Fruits, Vegetables, Bakery products	[17,21]
Alginate	Brown algae	Gel-forming, Film-forming, Gas barrier	Biodegradable, Biocompatible, Forms strong gels with Ca^2+^	Poor water barrier, Requires cross-linking for stability	Fruits, Vegetables, Minimally processed foods	[17,21]
Cellulose Derivatives (e.g., CMC, HPMC)	Plant cell walls	Film-forming, Viscosity modifier, Gas barrier	Good mechanical strength, Transparent, Water-soluble	Moderate water barrier, Limited antimicrobial activity	Fruits, Vegetables, Confectionery	[17,30]
**Proteins**						
Whey Protein	Milk	Excellent gas barrier (O_2_), Film-forming, Emulsifying	High nutritional value, Transparent, Good mechanical properties	Poor water barrier, Sensitive to moisture, Allergenic potential	Fruits, Vegetables, Nuts, Dairy products	[24,25]
Zein	Corn	Film-forming, Good mechanical strength, Moderate water barrier	Hydrophobic, Good adhesion, Biodegradable	Brittle films, Limited flexibility, High cost	Fruits, Nuts, Confectionery	[24]
Soy Protein	Soybeans	Film-forming, Emulsifying, Good mechanical strength	Abundant, Inexpensive, Biodegradable, Good oxygen barrier	Poor water barrier, Requires modification for optimal properties	Fruits, Vegetables, Meat alternatives	[24]
Gelatin	Animal collagen	Film-forming, Gel-forming, Good mechanical strength	Transparent, Flexible, Good oxygen barrier, Biodegradable	Poor water barrier, Animal-derived (religious/ethical concerns)	Fruits, Confectionery, Meat products	[24,26]
**Lipids**						
Waxes (e.g., Carnauba, Beeswax)	Plants, Bees	Excellent water barrier, Hydrophobic	Natural, Edible, Effective moisture control	Lack mechanical strength, Brittle, Can be opaque	Fruits, Vegetables (e.g., citrus, apples)	[15]
Essential Oils	Various plants	Antimicrobial, Antioxidant, Flavoring	Natural, Potent bioactive compounds, Aromatic	Volatile, Strong aroma, Can affect sensory quality, Low mechanical strength	Fruits, Vegetables, Spices	[15]

**Table 2:** Common nanomaterials and their specific functions in fruit preservation.

Nanomaterial Type	Specific Nanomaterial	Primary Function(s)	Mechanism of Action	Examples of Application	References
**Inorganic Nanoparticles**	Silver Nanoparticles (AgNPs)	Antimicrobial, Antifungal	Cell membrane disruption, protein denaturation, DNA damage	Fruits (e.g., strawberries, grapes), vegetables	[10,34]
	Zinc Oxide Nanoparticles (ZnONPs)	Antimicrobial, Antifungal, UV-blocking	ROS generation, enzyme inhibition, cell membrane damage	Apples, tomatoes, mangoes	[10,44]
	Titanium Dioxide Nanoparticles (TiO_2_NPs)	Antimicrobial, UV-blocking, Photocatalytic	ROS generation, degradation of organic compounds	Pears, peaches, leafy greens	[10]
	Copper Oxide Nanoparticles (CuONPs)	Antimicrobial, Antifungal	Release of Cu^2+^ ions, oxidative stress, protein damage	Avocados, bananas, citrus fruits	[35,45]
**Organic Nanofillers**	Cellulose Nanocrystals (CNCs)	Mechanical reinforcement, Gas barrier	Increased tortuosity, hydrogen bonding, dense network formation	Berries, apples, cucumbers	[30,37]
	Chitin Whiskers (ChWs)	Mechanical reinforcement, Antimicrobial	Enhanced film strength, interaction with microbial cell walls	Grapes, cherries, peppers	[38]
**Nanoemulsions/Nanoencapsulation**	Essential Oil Nanoemulsions	Antimicrobial, Antioxidant, Controlled release	Enhanced bioavailability, improved dispersion, protection from degradation	Citrus, apples, tomatoes	[40,46]
	Liposomal Nanocarriers	Encapsulation, Controlled release	Protection of active compounds, targeted delivery	Various fruits and vegetables	[42]

**Table 3:** Synergistic effects of combined functional additives in biopolymer-based nanocomposite edible coatings.

**Functional Additive 1**	**Functional Additive 2**	**Biopolymer Matrix**	**Observed Synergistic Effect**	**Reference**
Chitosan	Essential Oils (e.g., Clove)	Polysaccharide	Enhanced antimicrobial activity against a broader spectrum of pathogens; improved antioxidant capacity.	[66]
Alginate	Calcium Chloride (crosslinker)	Polysaccharide	Improved mechanical strength, reduced water solubility, enhanced barrier properties.	[67]
Starch	Lipids	Polysaccharide	Significantly reduced water vapor permeability; improved moisture barrier.	[68]
Whey Protein Isolate	Transglutaminase	Protein	Increased tensile strength, improved elasticity, enhanced water resistance.	[69]
Cellulose Nanocrystals	Chitosan	Polysaccharide	Enhanced mechanical properties (tensile strength, Young’s modulus); improved gas barrier.	[70]
Zinc Oxide Nanoparticles	Essential Oils (e.g., Thyme)	Various	Potentiated antimicrobial action; broader spectrum of activity; reduced effective concentration of EOs.	[71]
Plant Extracts (e.g., Green Tea)	Chitosan	Polysaccharide	Enhanced antioxidant activity; improved antimicrobial efficacy; synergistic delay in fruit ripening.	[72]

**Table 4:** Comparison of application methods for edible coatings: Advantages and Disadvantages.

Application Method	Advantages	Disadvantages	Suitability
**Dipping**	Simple, cost-effective, ensures complete coverage, suitable for irregular shapes.	Potential for excessive coating thickness, risk of contamination of dipping solution, less control over uniformity.	Small to medium scale operations, robust fruits/vegetables.
**Spraying**	Better control over coating thickness and uniformity, reduced material waste, adaptable to automation.	Requires specialized equipment (spray nozzles), potential for aerosol formation, and less effective for complex geometries.	Medium- to large-scale operations, delicate fruits/vegetables.
**Brushing**	Simple, low-cost, precise localized application.	Labor-intensive, less uniform coverage, not suitable for large-scale production.	Small-scale operations, specific localized treatments.
**Electrospinning**	Creates ultrafine fibers, high surface area, porous structure, controlled release of active compounds.	Complex equipment, high cost, low throughput, challenges in scalability.	Research and development, high-value products, specialized applications.
**Electrospraying**	Highly uniform and thin coating layers, even distribution of nanoparticles/nanoemulsions.	Similar to electrospinning in terms of complexity and cost, limited to liquid formulations.	Research and development, precise coating applications.
**Layer-by-Layer (LbL) Assembly**	Precise control over thickness and composition, multi-functional coatings, tailored properties.	Time-consuming, multi-step process, challenges in scalability for industrial application.	Research and development, highly customized coatings.

**Table 5:** Summary of recent studies (2023–2026) on nanocomposite coatings for specific fruits.

Year	Fruit/Vegetable	Biopolymer Matrix	Nanomaterial/Additive	Key Findings	Reference
2026	Strawberries	Chitosan	Self-assembled epigallocatechin gallate–cinnamaldehyde–L-arginine nanoparticles (ECL NPs)	The 0.2% ECL/chitosan coating suppressed visible spoilage during 6 days of storage, reduced weight loss to 28.49 ± 5.99%, and maintained fruit hardness at 95.05 ± 4.78 g. The nanoparticles also provided antioxidant and antibacterial activity.	[89]
2026	Mango (Harumanis)	Chitosan	Hydrophobic deep eutectic solvent nanoemulsion (HyDEN; menthol/thymol)	Chitosan–HyDEN showed high antioxidant activity and extended mango storage to 21 days, while better preserving weight, firmness, colour, TSS, acidity and antioxidant activity. It also provided postharvest anthracnose control.	[90]
2025	Strawberry	Carboxymethyl cellulose (CMC)	Chitosan nanoparticles, ZnO nanoparticles and chitosan–Zn complex nanoparticles	Nanoparticle-containing CMC coatings reduced postharvest deterioration. The CS/Zn-containing coatings maintained acceptable appearance, titratable acidity and ascorbic acid through day 6, with nanoparticle concentrations remaining below reported cytotoxic levels.	[91]
2025	Huangguan pear	Starch	Starch nanocrystals (SNCs) and cross-linked starch nanocrystals (CSNCs)	Both nanocomposite coatings improved preservation during 4 weeks of storage. The coating containing 10% CSNCs performed best, maintaining appearance, texture and biochemical quality and reducing weight loss and membrane permeability.	[92]
2024	Satsuma mandarin	Carboxymethyl cellulose (CMC)	2D layered double hydroxide nanosheets (LDH-NSs) + shellac	The nanocomposite improved coating wettability, adherence and barrier properties and prolonged postharvest shelf life while maintaining peel morphology, reducing weight loss and preserving hardness, colour and nutritional quality.	[93]
2024	Tomato	Chitosan oligosaccharide (COS)	Zinc oxide nanoparticles (ZnO NPs)	COS–ZnO nanocomposite coating formed a uniform film, significantly reduced water loss and skin wrinkling, improved the sugar–acid ratio, and enhanced vitamin C and carotenoid accumulation during postharvest storage.	[94]
2023	Banana	Chitosan	Cellulose nanocrystals (CNCs) + sodium tripolyphosphate (TPP)	The chitosan/CNC biocomposite coating substantially delayed deterioration and extended banana shelf life from 5 to 11 days.	[95]
2023	Mango (Kiett)	Alginate	Zinc oxide nanoparticles (ZnO NPs)	Alginate–ZnO NP coating reduced deterioration, controlled decay, maintained firmness and acidity, and moderated changes in respiration, weight loss, sugars and carotenoids during cold storage, thereby increasing mango storage life.	[44]

**Table 6:** Representative quantitative performance metrics for biopolymer-based edible coatings, with test conditions and units.

Parameter	Coating System	Value	Test Conditions/Units	Reference
WVTR	Pectin film	1120 ± 35	g m^−2^ d^−1^; standard conditions	[99]
WVP	Chitosan-HCl + PZNP + CNC	5.32 × 10^−10^	g m^−1^ Pa^−1^ s^−1^	[96]
OTR	LMP-casein + stearic acid (highest)	40.17	cc m^−2^ day^−1^	[97]
CO_2_TR	LMP-casein + stearic acid (highest)	34.87	cc m^−2^ day^−1^	[97]
CO_2_/O_2_ selectivity	Chitosan films	Higher than synthetic polymers	Ratio; humidity-dependent	[90]
Thickness	Pectin film	0.082 ± 0.004	mm	[99]
Thickness	Chitosan + ZnONPs	132–186	μm	[95]
Contact angle	Chitosan-HCl + PZNP + CNC	106.4	degrees; measurement time not specified	[96]
Tensile strength	Chitosan-HCl + PZNP	30.8	MPa	[98]
Tensile strength	Chitosan-HCl + PZNP + CNC	37.5	MPa	[96]
Elongation at break	Chitosan-HCl + PZNP	10.0	%	[71]
Elongation at break	Chitosan-HCl + PZNP + CNC	7.8	%	[96]
Antimicrobial (log reduction)	Chitosan on beef/mutton	1.95 (S. aureus); 1.07 (L. monocytogenes)	log_10_ CFU g^−1^; 21 days; 6.00 log_10_ CFU g^−1^ inoculum	[91]
Antioxidant (DPPH)	Chitosan/pullulan + carvacrol microcapsules	80.49	% scavenging	[100]
Antioxidant (ABTS)	Chitosan/pullulan + carvacrol microcapsules	89.40	% scavenging	[100]

**Table 7:** Regulatory status of common nanocomposite ingredients in edible coatings.

Ingredient	Type	Regulatory Status (e.g., FDA, EFSA)	Key Considerations/Limitations
Chitosan	Polysaccharide	Generally Recognized As Safe (GRAS) by FDA; Approved in EU for certain applications.	Purity, molecular weight, deacetylation degree.
Starch	Polysaccharide	GRAS by FDA; Approved in EU.	Source, modification methods.
Alginate	Polysaccharide	GRAS by FDA; Approved in EU as food additive (E401-E404).	Purity, heavy metal content.
Whey Protein	Protein	GRAS by FDA; Approved in EU.	Allergenicity, processing aids.
Carnauba Wax	Lipid	GRAS by FDA; Approved in EU as glazing agent (E903).	Purity, residual solvents.
Silver Nanoparticles (AgNPs)	Inorganic NP	Not explicitly GRAS; Requires premarket notification/authorization by FDA/EFSA.	Migration, size, concentration, toxicity.
Zinc Oxide Nanoparticles (ZnONPs)	Inorganic NP	Under review/case-by-case assessment by FDA/EFSA.	Migration, size, concentration, dissolution.
Titanium Dioxide Nanoparticles (TiO2NPs)	Inorganic NP	Approved as food additive (E171) in EU, but use restricted/under review for nano-form; FDA allows as color additive.	Size, aggregation, purity, potential genotoxicity.
Cellulose Nanocrystals (CNCs)	Organic Nanofiller	Under review/case-by-case assessment by FDA/EFSA.	Source, processing, purity, potential for aggregation.
Essential Oils	Plant Extract	GRAS by FDA for many; Approved in EU as flavorings.	Purity, concentration, potential for allergenicity, sensory impact.

**Table 8:** Comparative analysis of recent review papers on biopolymer-based nanocomposite edible coatings.

Feature	This Review	Singh et al., 2026 [108]	Tasnim & Islam, 2026 [83]	Dai et al., 2025 [109]	Culqui-Arce et al., 2026 [110]
**Publication Year**	2026	2026	2026	2025	2026
**Primary Focus**	Comprehensive: Mechanisms, Functional Additives, Safety, Comparative Analysis	Biopolymer-based edible coatings with integrated spoilage sensing and shelf-life extension functions	Active edible coatings for smart food preservation and sustainability	Application of chitosan and its derivatives in postharvest coating preservation of fruits	Polymeric Biocoatings for Postharvest Fruit Preservation: Advances, Challenges, and Future Perspectives
**Scope of Biopolymers**	Polysaccharides, Proteins, Lipids	Polysaccharides, Proteins	Polysaccharides, Proteins, Lipids	Chitosan and its derivatives	Polysaccharides, Proteins, Nanomaterials
**Nanomaterials Covered**	Inorganic NPs, Organic Nanofillers, Nanoemulsions/Encapsulation	Nanoparticles (general), Nanoemulsions	Nanoparticles (general), Nanoemulsions	Nanocomposites (general)	Nanomaterials (general)
**Depth of Mechanisms**	Detailed: Gas Barrier, Moisture, Antimicrobial, Antioxidant	Focus on spoilage sensing and shelf-life extension mechanisms	Focus on antibacterial, antioxidant, controlled-release systems, nano-enhancements	Focus on chitosan-based preservation mechanisms	General mechanisms of biocoatings
**Functional Additives**	Essential Oils, Plant Extracts, Probiotics, Prebiotics, Natural Crosslinkers	Limited focus on specific additives, more on sensing elements	Bioactive compounds, essential oils, nanoparticles, bacteriocins, natural antioxidants	Advanced modifications (e.g., multifunctional grafting)	Phenolic compounds, essential oils
**Safety & Regulatory**	Dedicated section: Migration, Regulatory Status (FDA, EFSA), Consumer Perception	Briefly mentioned	Briefly mentioned	Briefly mentioned	Briefly mentioned
**Application Methods**	Conventional & Emerging (Electrospinning, LbL)	General application methods	Novel manufacturing techniques (electrospraying, electrospinning, LbL)	Surface coating treatment (general)	Conventional and emerging application methods
**Nanoparticle-containing coatings as primary focus**	Yes—dedicated sections on inorganic NPs, organic nanofillers, nanoemulsions, and their migration/toxicity	No—nanomaterials are one component of a broader sensing-focused review	No—nanomaterials addressed within a general active-coating framework	No—chitosan and derivatives, with nanocomposites secondary	No—nanomaterials mentioned generally

**Table 9:** Techno-economic comparison of coating formation and application routes.

Route	Capital Intensity	Throughput	Dominant Energy Demand	Nanomaterial Utilisation Efficiency	Principal Scale-Up Barrier
Solution casting	Low	Batch	Solvent evaporation	Moderate; losses in coagulation bath	Batch variability; drying time
Melt extrusion	Moderate–high	Continuous	Heating and screw drive	High	Thermal degradation of actives
Dipping	Very low	High	Drying of residual water	Low; bath depletion and drag-out	Bath contamination; dose consistency
Spraying	Low	High	Droplet formation and drying	Moderate; overspray losses	Nozzle fouling; aerosol exposure
Electrospinning	High	Low	High voltage; solvent recovery	Moderate; fibre mat losses	Low throughput; solvent handling
Electrospraying	High	Low–moderate	High voltage; drying	High; low overspray	Formulation window narrow
Layer-by-layer	Very high	Low	Repeated drying between layers	High	Multi-step; long cycle time

## Data Availability

No new data were created or analyzed in this study. Data sharing is not applicable to this article.
